# Combinatorial sputtering of photoluminescent europium titanium oxide thin films†

**DOI:** 10.1039/d5ra04076k

**Published:** 2025-08-01

**Authors:** Junfeng Chen, Jeff Rao, Adrianus Indrat Aria

**Affiliations:** a Surface Engineering and Precision Centre, Faculty of Engineering and Applied Sciences, Cranfield University UK A.I.Aria@cranfield.ac.uk

## Abstract

Photoluminescent thin films were fabricated using a combinatorial physical vapour deposition (PVD) sputtering process, enabling rapid variation of europium oxide (Eu_2_O_3_) in titanium dioxide (TiO_2_) with concentrations varying from *x* = 0–1 in *x* = Eu/(Eu + Ti). Combinatorial sputtering enables synthesising samples with diverse compositions faster than traditional sol–gel, powder mixing, solvo/hydrothermal, and melt-quench processes. Post-heat treatment at 600 °C produced changes to the phase, structure and optical properties of the thin films. Scanning electron microscopy (SEM) revealed vertically oriented columnar microstructures in samples with concentrations lower than *x* = 0.5, exhibiting a narrower average columnar width of about 50 nm after annealing at 600 °C. X-ray diffraction (XRD) analysis indicated that TiO_2_ was in the anatase phase while Eu_2_O_3_ crystallises in a monoclinic structure. The nanocrystalline grain size exhibits noticeable changes after annealing. Fluorescence spectroscopy was used to study the photoluminescence of thin films. The excitation peak at 394 nm (^7^F_0_ → ^5^L_6_) measures spectral emissions, with the strongest emission at 613 nm (^5^D_0_ → ^7^F_2_).

## Abstract 2

Photoluminescent thin films were fabricated using a combinatorial physical vapour deposition (PVD) process, enabling rapid variation of europium oxide (Eu_2_O_3_) in titanium dioxide (TiO_2_) with concentrations varying from *x* = 0–1 in *x* = Eu/(Eu + Ti).

## Graphical abstract

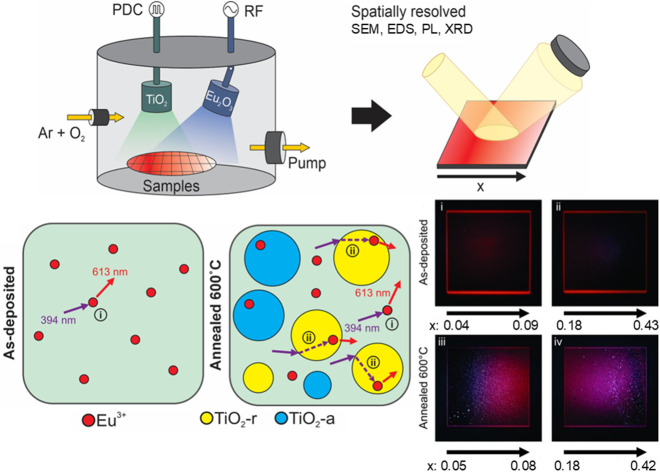

## 1 Introduction

Functional thin films are essential in many engineering applications ranging from antireflective films to multi-barrier OLEDs.^1–4^ These thin films contain compounds with two or more constituent components, where the ability to vary the composition plays a pivotal role in optimising their properties and performance. For instance, the optical and dielectric properties of compound semiconductor thin films are reported to be highly dependent on the ratio of their constituent components.^5,6^ The deployment of rapid or high-throughput methodologies such as combinatorial synthesis, whether through liquid or gas phase chemistries provides a significant advantage over traditional fabrication methodology. For example, combinatorial methods have been utilised for the rapid fabrication and evaluation of 2478 quinary alloys composed of aluminium and various transition metals.^7^ This approach underscores the capability of combinatorial sputtering to efficiently produce large quantities of thin films with diverse structural and compositional profiles while also enabling precise control over film thickness. Combinatorial synthesis enables broad compositional variation and the exploration of diverse properties in a minimal number of experiments. Consequently, this method has been widely adopted for the development of photoluminescence (PL) thin films and battery materials.^8,9^

Materials with PL properties are functional materials that absorb photon energy from external sources and subsequently re-emit that energy as photons with different energy levels. They are used in optoelectronic applications, ranging from lightings and displays to photovoltaics and sensors. Examples of PL materials include quantum dots (QDs) and fluorescent conjugated polymers. Amongst these materials, halide perovskite colloidal QDs are the most commonly used in PV and display applications because of their cost-effectiveness and diverse wavelength-shifting characteristics.^10,11^ However, the limitations associated with these materials include reduced stability and minimal PL quantum yield (PLQY).^12^ In this study, europium titanium oxide (ETO) was selected as a model system offering advantages in terms of intense luminescence with a potentially high intrinsic PLQY of 91%,^13^ making this material system attractive to potential applications in PL and photocatalysis (PC).^14,15^ Eu_2_O_3_ is a wide bandgap semiconductor that has typically a sharp red emission at 613 nm under UV excitation resulting from the ^5^D_0_ → ^7^F_2_ transition from Eu^3+^ ions, making it ideal for applications requiring high colour purity and PL intensity.^16–18^ However, Eu^3+^ ions exhibit a narrow absorption cross-section, limiting their excitation efficiency.^18^ TiO_2_ is a wide-bandgap semiconductor that exhibits prominent crystalline polymorphs, including rutile, anatase, and brookite, with bandgap values ranging from 3.0 to 3.3 eV.^19^ Anatase and rutile are commonly synthesised under atmospheric pressure and temperatures between room temperature and 1200 °C.^20,21^ These films remain amorphous up to 350 °C, but undergo phase transformations beyond this temperature, resulting in distinct crystalline structures.^22^ TiO_2_ is also known to frequently contain defect states such as oxygen vacancies, that introduce intermediate energy states within its bandgap.^23,24^ These defect states can facilitate energy transfer to nearby Eu^3+^ ions when in proximity to Eu_2_O_3_, thereby enhancing PL emission.^25^

Despite being widely reported in the literature, no unified understanding of the relationship between luminescence and physicochemical properties has been established. Several studies suggest optimal luminescent properties are achieved in crystalline titanium oxide with low doping concentrations of Eu.^26^ For example, TiO_2_ thin films with a Eu concentration of 0.2 at% are reported to show significant structural modifications, such as an increase in crystallite size of the anatase TiO_2_ when annealed at 800 °C.^27^ The incorporation of Eu^3+^ ions within the thin film matrix is essential for inducing PL. The thin films in this study exhibit variable concentrations of Eu and Ti, with compositions defined by the ratio *x* = Eu/(Eu + Ti). However, as the concentration of Eu increases, larger voids tend to form within the film, adversely affecting the PL performance of the Eu^3+^ ions. These voids act as non-radiative recombination sites, quenching the PL by facilitating energy loss through non-radiative pathways rather than emission.^28^ In other studies argue that *x* = 0.1 yields optimum PL properties.^29^ Moreover, evidence of PL in amorphous ETO thin films remains elusive.^30^ Further research is needed to elucidate the specific structural requirements for enhancing PL performance. In this study, rapid screening by combinatorial sputtering is utilised to determine how differing Eu concentrations are linked to changes in PL intensities.

Here, we employ combinatorial gas-phase magnetron sputtering at room temperature to elucidate the interrelationship between the composition, crystallography, and PL properties of ETO thin films. Combinatorial sputtering enables the simultaneous deposition of two or more sputtering targets, creating a region where deposition fluxes overlap and thereby allowing the controlled synthesis of composite films with spatial variations in elemental composition in each deposition run.^7^ Combinatorial sputtering facilitates the fabrication of thin films with varying compositions without the complexity of gas- or liquid-phase chemical reactions.^22,31^ This makes it particularly advantageous for rapid materials discovery and optimisation, enabling the screening of a broad range of material properties with fewer experiments. While this technique represents a pioneering approach for investigating ETO thin films compared to traditional methods such as sol–gel and powder mixing,^18,29,32–40^ it is less suitable for large-scale production due to the intentional spatial heterogeneity of the deposited thin films. Sol–gel and melt-quench methods, which are more suitable for batch processing, rely on liquid precursors that may introduce unintentional contamination. Powder mixing methods, while simple and economical, are unsuitable for thin film fabrication and may introduce phase separation from polydispersity and heterogeneous mixing. Once an optimal composition is identified by combinatorial sputtering, it can be transferred to conventional thin film deposition techniques such as pulsed laser deposition (PLD), thermal/e-beam evaporation, and chemical vapour deposition (CVD) that offer greater scalability and uniformity for industrial applications.^31,40^

In this study, we focus on combinatorial sputtering to vary the composition of *x* = Eu/(Eu + Ti) with *x* = 0–1 within a single deposition and employ post-deposition annealing to induce crystallisation. While other film characteristics and environmental factors may also play a role, this study specifically investigates the impact of compositions and annealing on the crystallography and PL properties of the ETO thin films. The methodology reported herein provides a robust platform for tailoring PL properties through controlled deposition and post-deposition processing. The high-throughput combinatorial approach employed here facilitates the rapid evaluation of thin film properties and performance, enabling accelerated optimisation for specific applications.^41,42^ This technique can be further applied to systematically optimise PL properties across a broad spectrum of luminescent thin films beyond ETO, such as Al_2_O_3_–Y_2_O_3_ (YAlO) thin films.^41^ Additionally, this work establishes a model framework for the strategic development of functional thin films, particularly suited for optical applications with stringent compositional requirements such as solar photovoltaics and electronic displays.

## 2 Methodology

### 2.1 Combinatorial sputtering

Combinatorial sputtering thin film deposition was carried out using two magnetrons in a confocal arrangement, with a Eu_2_O_3_ target (purity: 99.9%, K-tech supplies) and a TiO_2_ target (purity: 99.9%, K-tech supplies) in a 5% oxygen/95% argon gas mix. Both sputtering targets have dimensions of 76.2 mm diameter and 3.18 mm thick placed on 3 mm thick Cu backing plates using indium bonding.

**Fig. 1:**
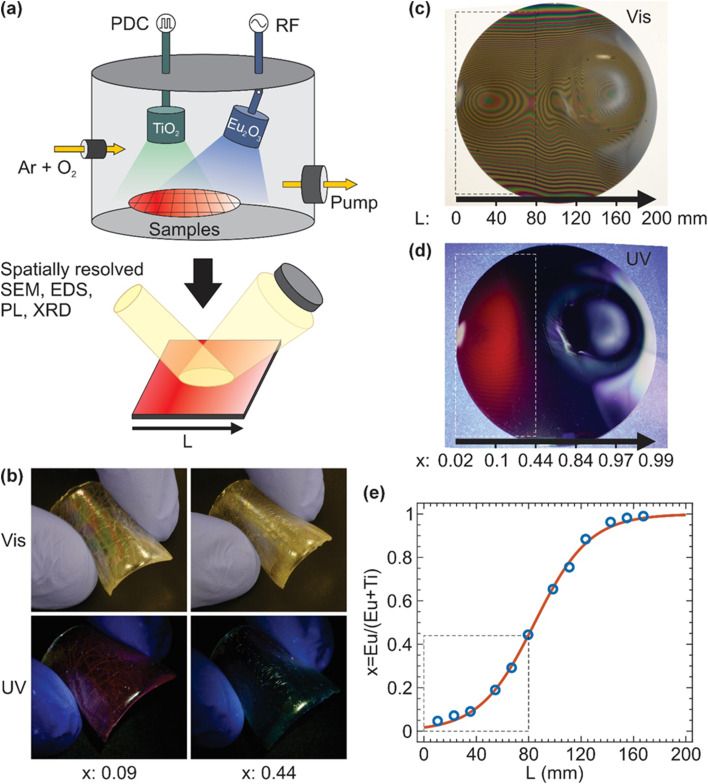
(a) Schematic of the reactive combinatorial sputtering setup, comprising two magnetrons in a confocal arrangement, with the coating produced in an O_2_/Ar mixture. TiO_2_ and Eu_2_O_3_ were simultaneously sputtered with pulsed DC at a power density of 2.19 W cm^−2^ and RF at a power density of 1.53 W cm^−2^, respectively. The thin films were then spatially characterised by SEM/EDS, PL, XRD, and UV/Vis before and after annealing at 600 °C. *L* is defined as the relative lateral position along the substrate measured from the TiO_2_ to the Eu_2_O_3_ target, where a lower *L* corresponds to a position closer to the TiO_2_ target and a higher *L* corresponds to a position closer to the Eu_2_O_3_ target. (b) Photographs of the coating with *x* = 0.09 and *x* = 0.44, are deposited on PDMS, which is then flexed and exposed to visible and UV light. Photographs of the as-deposited ETO coating on a 200 mm Si wafer under (c) visible and (d) 365 nm UV light. The Eu/(Eu + Ti) composition varies from *x* = 0.01 to *x* = 1 as *L* increases from 1 to 200 mm. (e) The relationship of the composition *x* as a function of *L* in Eu/(Eu + Ti). The solid line is a logistic regression fitting of *x* as a function of *L*, where *x* = 0.01 at *L* = 0 mm and *x* = 0.99 at *L* = 200 mm. The areas inside the dashed boxes in (c)–(e) indicate the area with the most visible luminescence between *x* = 0.01 and *x* = 0.44.

The targets are positioned 80 mm above the substrates. The magnetron with the TiO_2_ target was facing straight down with 0° tilt angle, while that with the Eu_2_O_3_ target was tilted 15° towards the centre of the substrate table, with the distance between the centres of the two magnetrons being 140 mm (Fig. 1a). The substrate was placed under both targets, with *L* defined as the relative lateral position along the substrate measured from the TiO_2_ to the Eu_2_O_3_ target. A lower *L* value corresponds to a position closer to the TiO_2_ target, while a higher *L* value indicates proximity to the Eu_2_O_3_ target. The centre of the TiO_2_ target corresponds to *L* = 30 mm, and the centre of the Eu_2_O_3_ target corresponds to *L* = 170 mm. This positional parameter reflects the compositional variation formed across the substrate during sputtering.

Before sputtering, the chamber was evacuated using a turbomolecular pump to a base pressure of 2.0 × 10^−6^ torr. TiO_2_ was sputtered by pulsed direct-current (DC) power supply at 250 kHz and 1616 ns pulse width, and Eu_2_O_3_ by radio frequency (RF) power supply at 13.56 MHz. The sputtering was done at a total O_2_/Ar mix flow rate of 20 sccm with a process pressure of 1 × 10^−2^ torr. Eu_2_O_3_ was sputtered with power densities of 1.53 W cm^−2^ and TiO_2_ was deposited with power densities of 2.19 W cm^−2^. The thickness of the ETO thin films was maintained within 3–4 μm to ensure sufficient material for accurate and reliable XRD measurements while minimising internal stress that may lead to cohesive failure and delamination.

A batch of samples was produced in a single deposition run. Each sample was subsequently sectioned at the centre, with one half retained for direct characterisation in the as-deposited state, and the other half subjected to annealing in air at 600 °C for one hour using a Carbolite ELF11/14 furnace prior to characterisation. For annealing, the heat-up rate is 2 °C min^−1^ with unforced natural cool-down for 6 hours until room temperature.

### 2.2 Substrate materials

The ETO thin films were deposited on two types of substrates 25 × 25 × 1 mm quartz (Agar scientific) and a 25 × 25 × 0.5 mm Si wafer cut from 100 mm diameter Si wafer (〈100〉, p-doped, Si-Mat Silicon Materials). The substrates were cleaned prior to deposition in ultrasonic baths of acetone followed by ultrasonic baths in IPA for 10 minutes each. Furthermore, the substrates are exposed to a 50 W oxygen plasma for 3 minutes to increase the surface energy of substrates for better coating adhesion.

During each sputtering process, four identical substrates are used. One substrate is positioned directly beneath the TiO_2_ target, while another is placed directly under the Eu_2_O_3_ target. The remaining two substrates are positioned equidistantly between the two targets. Further deposition was performed on a 200 mm diameter Si wafer (〈100〉, p-doped, Pi-Kem) positioned on the substrate table to effectively cover the entire intersectant sputtering area between the two targets. This approach provides a systematic method for assessing the PL characteristics of the thin films and correlating them with the distribution between Eu_2_O_3_ and TiO_2_.

Deposition of ETO thin film coating was also performed on flexible substrates of polydimethylsiloxane (PDMS) measuring 25 × 25 × 1 mm, without annealing, allowing a comparison between the thin films deposited on quartz and Si wafer. A mixture of 9 g of silicone elastomers base (SYLGARD™) and 1 g of curing agent (SYLGARD™) was prepared to form a PDMS solution. The solution was subsequently cured at 80 °C for 1 hour, resulting in the formation of a flexible material.

### 2.3 Characterisation

The photographs of the samples were taken during exposure to visible light and UV LED (LZ1-10UV0R VIOLET LED 365 nm). Before fluorescence measurements, calibration with deionised (DI) water is required. A 200 mm diameter silicon wafer was positioned between two magnetrons for the deposition of ETO thin films, facilitating the observation of fluorescent properties of ETO thin film with *x* = 0–1 when exposed to visible and UV light (Fig. 1c and d). PL was carried out by HORIBA Scientific, Jobin-Yvon SAS, Fluoromax+ spectrofluorometer with a 150 W xenon lamp as the excitation light source with a spot size of 10 × 5 mm. Measurements were taken at the centre of each sample, with the surrounding area masked using non-reflective black metal to minimise PL emission from the masked region. The emission spectra are measured from 550 to 710 nm with an increment of 1 nm, a slit of 1 nm, and 0.1 s dwell time while fixing the excitation wavelength at 394 nm with a 1 nm slit width.

Scanning electron microscopy (SEM) coupled with energy dispersive X-ray (EDS) were used to determine the microstructure and elemental compositions of ETO films. SEM imaging was carried out using TESCAN S8000 SEM with a 10 keV acceleration voltage, 40k magnification, and a 5 mm working distance. Quantitative elemental analysis was performed using EDS along the substrate within the unmasked central region of each sample, corresponding to the area where PL measurements were taken. Here, the film composition is defined as *x* = Eu/(Eu + Ti), where Eu and Ti are the measured atomic compositions (at%) of both elements, as determined by EDS. The elemental EDS analysis was carried out using an Oxford Instruments EDS system (Ultim Max Silicon Drift Detector EDS) with a 20 keV acceleration voltage, 40k magnification, and a 6 mm working distance, corresponding to a scanned area of 7 × 9.5 μm. The elemental composition at each measurement point was quantified using EDS area mapping of the film cross-section, with the counts averaged over the entire scanned area. Unless specified otherwise, the quantitative elemental composition reported herein reflects the spatial variations in the lateral direction while averaging out any variations across the film thickness. The EDS area maps were collected and quantified using Oxford Instruments Aztec software.

XRD was carried out to examine the crystallography of the films by SIEMENS D5005 powder XRD using 40 kV, 40 mA and wavelength 0.154 nm Cu Kα radiation source with Ni-filter. The scanning range of 2*θ* is from 10° to 90°, with 4001 seconds, step size: 0.02 2*θ* s^−1^, spot width: 17.5 mm, spot length: 5.2–25.5 mm, slit: 2–2–0.6 mm. The transmittance and bandgap were determined by UV-Vis spectroscopy.^43^

## 3 Results

### 3.1 Europium titanium oxide thin films deposition

ETO Thin films were deposited on flexible PDMS substrates to compare their PL properties with those deposited on hard substrates, such as quartz and Si wafers. The coatings on PDMS had a composition of *x* = 0.09 and *x* = 0.44, and all samples were analysed in their as-deposited state without annealing. The as-deposited thin films on PDMS exhibited differing behaviour when exposed to visible and 365 nm UV light (Fig. 1b and SI 19†). At the *x* = 0.09, the ETO film exhibited strong PL under 365 nm UV light, exhibiting luminescence even when the substrate is slightly flexed. However, the amorphous nature of the thin films at *x* = 0.44 prevents PL under similar conditions, suggesting that at this composition the PL activity is suppressed in the as-deposited state.

To further understand the effect of film composition on the PL behaviour, an ETO thin film was deposited on a 200 mm Si wafer (Fig. 1c and d). Under visible light, the coated wafer exhibited fringes due to spatial variations in film thicknesses ranging between 3 to 4 μm (Fig. 1c). Such variations in thickness lead to constructive and destructive interference of the light reflected by the coating and the underlying substrate. The area under the TiO_2_ target at *L* = 30 mm from the left edge of the wafer has the highest thickness due to the higher deposition rate of TiO_2_. When illuminated at 365 nm (Fig. 1d), the left-hand portion of the wafer between *L* = 5 and 80 mm from the left edge of the Si wafer exhibits red PL emission, with the brightest area being around *L* = 35 mm. In contrast, the right-hand portion of the wafer at *L* > 90 mm exhibited no PL emission (Fig. 1d).

The spatially measured composition (Fig. SI 1–8†) of the ETO thin film is defined herein as *x* = Eu/(Eu + Ti), which varies between *x* = 0.01 and *x* = 0.99 across the 200 mm diameter of the wafer (Fig. 1e). As experimentally measured by EDS, the ETO film under the TiO_2_ target, at approximately *L* = 30 mm, comprises of 3 at% Eu, 31 at% Ti, and 66 at% O, corresponding to *x* = 0.09. The film near the position equidistant from both TiO_2_ and Eu_2_O_3_ targets, at around *L* = 98.5 mm, exhibits an elemental composition of 20 at% Eu, 11 at% Ti, and 69 at% O, corresponding to *x* = 0.65. At *L* = 170 mm, which is under the Eu_2_O_3_ target, the film consists of 27 at% Eu, 1 at% Ti, and 72 at% O, corresponding to *x* = 0.96. The compositional relationship between *x* = Eu/(Eu + Ti) and the position *L* (in mm) from the left edge of the wafer can be approximated by the following logistic regression (eqn (1)):1
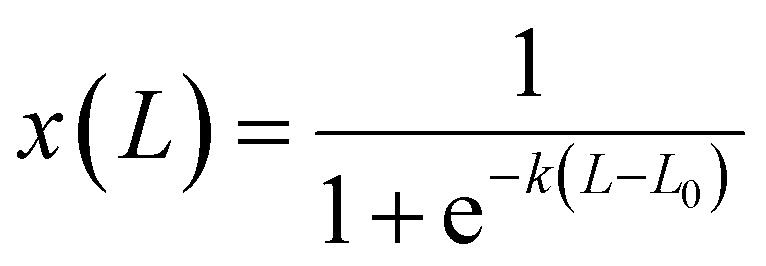
with *L*_0_ is the inflection position (84.66 ± 1.59 mm) and *k* is the curve steepness (0.048 ± 0.003). Both *L*_0_ and *k* are highly dependent on the sputtering parameters of each target, including the target materials, applied power, sputtering mode (*e.g.* PDC, RF), local magnetic field of the magnetron, and gas composition near the target. Therefore, the relationship between *x* and *L* can be further tuned by independently varying the sputtering parameters of each target.

The photographs of the ETO thin films, captured using a consumer-grade digital camera, highlight the regions with the highest PL emission intensity at *L* = 5–60 mm (Fig. 1c), corresponding to *x* = 0.01–0.25 (Fig. 1d). Although the area between *L* = 60–80 mm, corresponding to *x* = 0.25–0.4, also exhibits PL emission, its intensity is too weak to be effectively captured by the camera. PL emissions in this region could only be observed using fluorescence spectroscopy (Fig. 1c–e, marked by a dashed box).

### 3.2 Photoluminescent properties

**Fig. 2:**
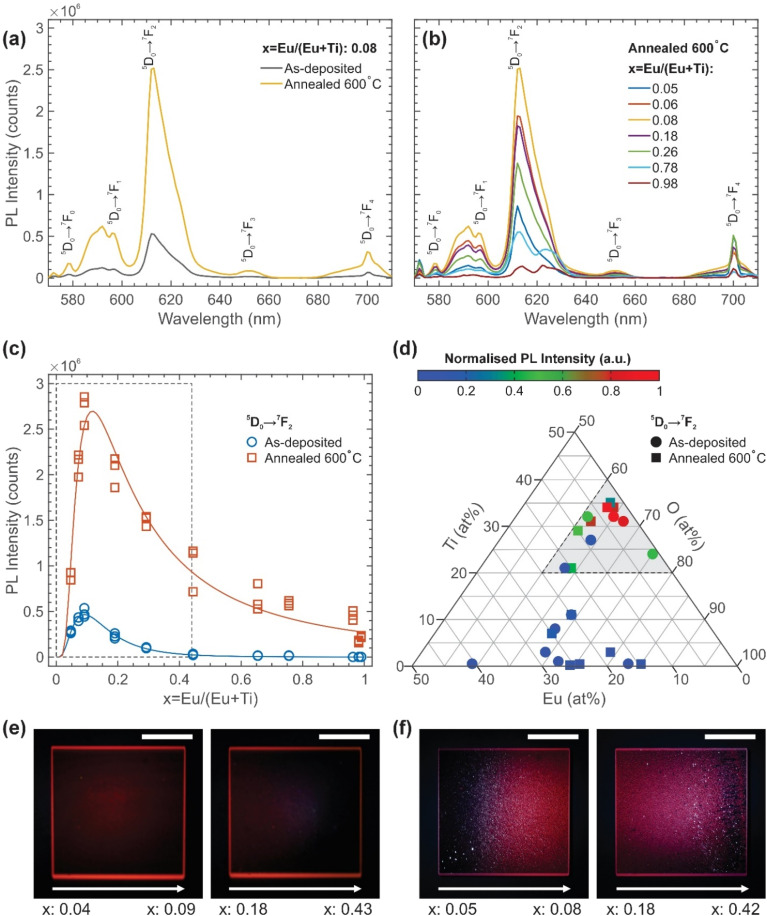
(a) The emission spectra measured over a wavelength range of 550–710 nm for a film having a composition where *x* = 0.08 Eu/(Eu + Ti), as-deposited and after annealing at 600 °C using 394 nm UV excitation, corresponding to ^7^F_0_ → ^5^L_6_ excitation. (b) Emission spectra over a wavelength range of 550–710 nm with changes in composition where *x* = 0.05, 0.06, 0.08, 0.18, 0.26, 0.78 and 0.98 Eu/(Eu + Ti), after annealing at 600 °C using 394 nm UV excitation, corresponding to ^7^F_0_ → ^5^L_6_ excitation. (c) The changes in PL emission intensity of the ^5^D_0_ → ^7^F_2_ transition with compositions between *x* = 0.01 to 1 Eu/(Eu + Ti), using 394 nm excitation, as-deposited and after annealing at 600 °C. (d) Normalised PL emission intensities of ^5^D_0_ → ^7^F_2_ transition for both as-deposited and annealed films as a function of the ternary elemental plot of Eu, Ti and O concentration as recorded by EDS. The areas inside the dashed box in (c) and the shaded triangle in (d) indicate the composition with the most visible luminescence between *x* = 0.01 and *x* = 0.44. (e) Photographs display the thin films with *x* ranging from 0.04 to 0.09 and from 0.18 to 0.43 on quartz as-deposited under 365 nm UV light. Scale bars in photographs are 1 mm. (f) Photographs display the thin films with *x* ranging from 0.05 to 0.08 and from 0.18 to 0.42 on quartz annealed at 600 °C under 365 nm UV light. Scale bars in photographs are 1 mm.

The PL emission spectra of ETO thin films exhibit multiple peaks at a wavelength between 550–710 nm that can be associated with a radiative transition from an excited state band edge ^5^D_0_ to the ^7^F_*J*_ (*J* = 0–4) ground state of Eu^3+^.^34^ Under 394 nm UV excitation, corresponding to ^7^F_0_ → ^5^L_6_ excitation, the as-deposited ETO films exhibited PL emission with the dominant PL peak at 613 nm and attributed to the ^5^D_0_ → ^7^F_2_ transition (Fig. 2a, SI 14 and 16†).^18^ Additional emission peaks at 579 and 700 nm, attributed to ^5^D_0_ → ^7^F_0_ and ^5^D_0_ → ^7^F_4_, respectively, their PL intensities were much weaker than that of ^5^D_0_ → ^7^F_2_ transition.^18,29,32^

The intensity of the emission spectra was found to be highly influenced by the annealing process (Fig. SI 15†). At a composition where *x* = 0.08, annealing at 600 °C led to an increase in the intensity of all transition peaks attributed to ^5^D_0_ → ^7^F_*J*_ (*J* = 0–4) (Fig. 2a). A transition peak at 653 nm became more obvious after the annealing and is attributed to the ^5^D_0_ → ^7^F_3_ transition. Furthermore, after annealing, the intensity of ^5^D_0_ → ^7^F_2_ transition at 613 nm was observed to be five times higher than that of the as-deposited films. As this transition exhibits the strongest PL emission peak after annealing, it is used here to quantify the effects of annealing on the PL emission intensity.

The PL spectra were also found to be highly dependent on the film compositions (Fig. 2b). The intensity of the ^5^D_0_ → ^7^F_2_ transition at 613 nm varies strongly with the change in film composition.^44,45^ Although weaker, the intensity of the remaining peaks was also observed to vary with the change in film composition. Note that the intensity of peaks for ^5^D_0_ → ^7^F_0_, ^5^D_0_ → ^7^F_1_, ^5^D_0_ → ^7^F_2_, and ^5^D_0_ → ^7^F_3_ transitions reached a maximum at a composition where *x* = 0.08, and the relationship between intensity and composition at these transitions are the same as the ^5^D_0_ → ^7^F_2_ transition. In contrast, the PL intensity of the ^5^D_0_ → ^7^F_4_ transition reached a maximum at *x* = 0.26.

To further understand the effect of Eu^3+^ concentration on the PL emission intensity, we quantified the PL emission intensity of ^5^D_0_ → ^7^F_2_ transition as *x* increased from 0.02 to 0.99 (Fig. 2c). For the as-deposited film, the PL emission intensity increased rapidly with increasing Eu^3+^ concentration when *x* was increased from 0.02 to 0.08, reaching a maximum intensity at *x* = 0.08. Further increases in *x* from 0.08 to 0.44 resulted in a reduction of PL intensity. As stated earlier, films deposited on PDMS, Si, and quartz substrates with compositions in the range of 0 < *x* < 0.44 (Fig. 2c, area marked by a dashed box) exhibited red emission, visible to the naked eye when exposed to 365 nm excitation (Fig. 2e). However, such red luminescence was barely visible for 0.43 < *x* < 0.44 (Fig. 2e). When the composition *x* > 0.44, the PL emission could no longer be observed as the intensity fell below the sensitivity of the PL detector. The overall PL intensity of the as-deposited films can be approximated by a Lognormal distribution function having a maximum when the composition is *x* = 0.08 (eqn (SI 5)†).

A significant increase in PL intensity was observed after the films had been annealed, with the PL intensity of ^5^D_0_ → ^7^F_2_ transition increasing by ∼5× than that of the as-deposited films. For the annealed film, the PL intensity increased rapidly with increasing *x* from 0.02 to 0.08. ETO thin films with these compositions exhibited strong red luminescence clearly visible to the naked eye under 365 nm excitation (Fig. 2f). Further increases in *x* from 0.08 to 0.44 resulted in a reduction in the intensity, although the red luminescence was still visible to the naked eye (Fig. 2f). In addition, the PL spectra of the thin films with compositions *x* > 0.44 could not be observed in their as-deposited state. However, these became more significantly pronounced following the annealing process. This transformation suggests that annealing not only enhances the crystallinity and structural ordering of the films but also activates or amplifies their PL characteristics. The overall PL intensity of annealed films can be approximated by a Weibull distribution function with a maximum at a composition where *x* = 0.08 (eqn (SI 6)†). Nevertheless, the overall relationship between intensity and composition remains the same, *i.e.* rapid increase with intensity as *x* increases from 0.01 to 0.08 and decrease slowly as *x* increases further from 0.08 to 1.

In terms of the ternary elemental composition, films with 0 < *x* < 0.44 correspond to the following elemental ratios: Eu at 0–20 at%, Ti at 20–40 at%, and O at 60–80 at%, for both as-deposited and annealed states (Fig. 2d, shaded triangle). The regions exhibiting the highest PL emission intensities in the ETO thin films are indicated in red, with slightly weaker emission intensities marked in green, these regions are confined within a shaded triangular area. Films with compositions falling outside these ranges exhibited weak or negligible red emission under UV illumination. Notably, both as-deposited and annealed thin films showed peak PL emission intensity at approximately *x* = 0.08, consistent with prior studies identifying optimal PL performance in the 7–10 at% Eu/(Eu + Ti) range.^18,29^ Furthermore, annealing was observed to influence the O content in the films, with variations in O concentration either increasing or decreasing post-annealing. This suggests that the PL emission intensity of ETO thin films is highly dependent on their elemental composition, particularly the Eu content. Precise control of the elemental composition is critical for achieving strong PL properties. This highlights the important role of both composition and post-deposition annealing in optimising the performance of ETO thin films for potential applications in luminescent devices.

### 3.3 Crystallography and microstructures

**Fig. 3:**
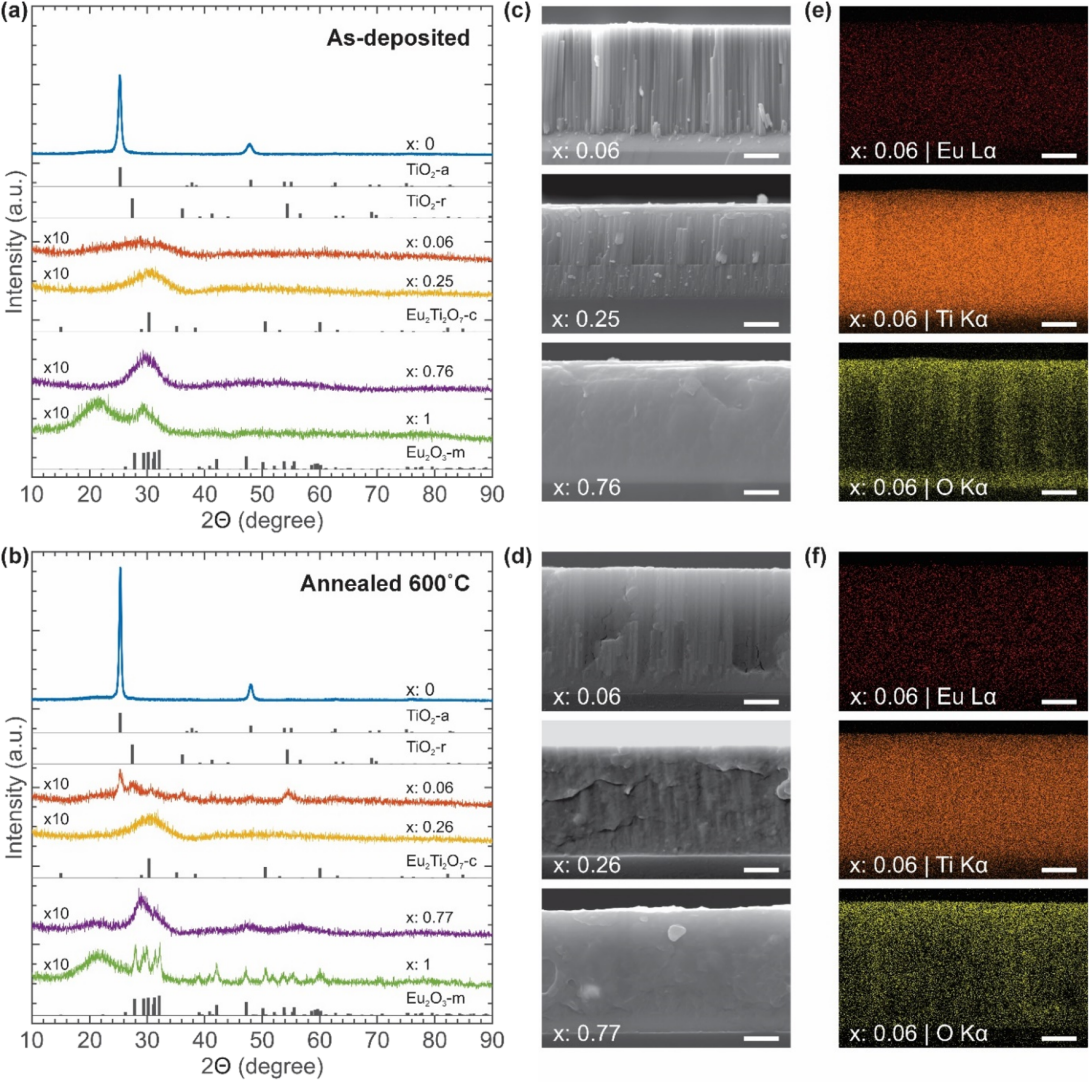
Powder XRD patterns for the (a) as-deposited ETO thin films with *x* = 0, 0.06, 0.25, 0.76 and 1 on the quartz substrates and (b) ETO thin films annealed at 600 °C with *x* = 0, 0.06, 0.26, 0.77 and 1 on the quartz substrates. TiO_2_-a: anatase TiO_2_ (ICSD 202242) TiO_2_-r: rutile TiO_2_ (ICSD 193266), c-Eu_2_Ti_2_O_7_: cubic Eu_2_Ti_2_O_7_ (ICSD 196405), m-Eu_2_O_3_: monoclinic Eu_2_O_3_ (ICSD 631461). SEM cross-section images of the (c) as-deposited ETO thin films with *x* = 0.06, 0.25 and 0.76 and (d) ETO thin films after annealing at 600 °C with *x* = 0.06, 0.26 and 0.77. Corresponding Eu, Ti, and O elemental EDS maps for the (e) as-deposited ETO thin films with *x* = 0.06 and (f) ETO thin films after annealing at 600 °C with *x* = 0.06. The elemental quantification from EDS maps is used to calculate *x*. Scale bars of the SEM and EDS images are 2 μm.

The XRD pattern of the as-deposited film with *x* = 0, *i.e.* TiO_2_ film, showed crystalline structures with peaks at 2*θ* of 25.32° and 47.98°, which are attributed to the 〈101〉 and 〈200〉 plane from anatase TiO_2_ (space group: *I*4_1_/*amd*), respectively with *a* = 0.3805 nm, *c* = 0.942 nm, and crystallite size of 22.7 nm (eqn (SI 2)† and Fig. 3a). As *x* increases to 0.06, the as-deposited films become fully amorphous without any discernible peaks (Fig. 3a). Despite being amorphous, the film exhibited vertically oriented columnar microstructures with an average column width of around 100 nm (Fig. 3c). EDS elemental mapping reveals a uniform distribution of Eu and Ti throughout the entire thickness of the ETO thin films, indicating the spatial co-location of Eu within the amorphous TiO_2_ matrix (Fig. 3e). However, the O map exhibits slight spatial nonuniformity, with an O-deficient zone observed at the base and middle section of the film.

As *x* increased to 0.25, a very broad peak formed at 2*θ* of ∼30° (Fig. 3a). This broad peak might be attributed to cubic Eu_2_Ti_2_O_7_ (space group: *Fd*3̄*m*, 227) or monoclinic Eu_2_O_3_ (space group: *C*2*m*, 12), suggesting the early formation of Eu_2_Ti_2_O_7_ and/or Eu_2_O_3_ nanocrystallites. While this film also exhibited vertically oriented columnar microstructures with an average column width of around 100 nm, the columns were less distinctive (Fig. 3c). As *x* is further increased to 0.76, a broad peak at 2*θ* of ∼30°, like that on the *x* = 0.25 film (Fig. 3a), became more pronounced. This suggests further growth of Eu_2_Ti_2_O_7_ and/or Eu_2_O_3_ nanocrystallites. This film no longer exhibited columnar microstructures. Instead, it exhibited a fine-grained smooth microstructure with no distinguishable features (Fig. 3c). At this point, the film was no longer exhibiting any red luminescence. As a comparison, the XRD pattern of as-deposited film with *x* = 1, *i.e.* Eu_2_O_3_, also showed a broad peak at 2*θ* of ∼30°, similar to that on the *x* = 0.76 film (Fig. 3a). The additional broad peak observed at 2*θ* of ∼20° could be attributed to the quartz substrates (Fig. SI 9†).

The XRD pattern of the annealed TiO_2_ thin film (*x* = 0) shows prominent crystalline characteristics comparable to the as-deposited film, with pronounced diffraction peaks at 2*θ* values of 25.32° and 47.98°. These peaks correspond to the 〈101〉 and 〈200〉 planes of the anatase TiO_2_ phase (space group: *I*4_1_/*amd*), respectively. Lattice parameters are calculated as *a* = 0.3789 nm and *c* = 0.943 nm. The crystallite size of the annealed film is determined to be 58.4 nm, indicating a significant increase relative to the as-deposited film (eqn (SI 2)† and Fig. 3a).

As *x* increases to 0.06, peaks forming at an annealing temperature of 600 °C suggest the formation of crystalline clusters, corresponding to the anatase phase of TiO_2_ and nano-sized rutile TiO_2_ clusters. The composition ratio between the anatase and rutile phases is determined to be 68 : 32 (Fig. 3b). The anatase TiO_2_ phase exhibits lattice parameters of *a* = 0.3809 nm and *c* = 0.945 nm with a crystallite size of 31.8 nm, while the rutile TiO_2_ phase exhibits lattice parameters of *a* = 0.4623 nm and *c* = 0.297 nm with a crystallite size of 42.4 nm (eqn (SI 2)†). This is in contrast to the as-deposited *x* = 0.06 ETO film where it exhibits an amorphous XRD pattern without any discernible peaks. Notably, this film displays vertically oriented columnar microstructures with an average column width of approximately 50 nm (Fig. 3d). EDS elemental mapping demonstrates a homogeneous distribution of Eu, Ti, and O across the entire film thickness, confirming that Eu remains uniformly dispersed within the TiO_2_ matrix without detectable elemental segregation despite the crystallisation of the TiO_2_ into anatase and rutile phases during annealing (Fig. 3f). Note that the spatial distribution of O becomes more uniform after annealing and the O-deficient zone at the base of the columnar structure is no longer present.

As the Eu content increased to *x* = 0.26, a broad peak began to emerge around 2*θ* ∼ 30° (Fig. 3b). This broad peak may correspond to either the cubic Eu_2_Ti_2_O_7_ phase (space group: *Fd*3̄*m*, 227) or the monoclinic Eu_2_O_3_ phase (space group: *C*2*m*, 12), indicating the initial formation of Eu_2_Ti_2_O_7_ and/or Eu_2_O_3_ nanocrystallites. Additionally, while this film displayed vertically oriented columnar microstructures with an average column width of approximately 50 nm, the distinction between these columns was less pronounced (Fig. 3d). As *x* increased further to 0.77, the broad peak at 2*θ* ∼ 30°, observed in the *x* = 0.26 film (Fig. 3b), became slightly sharper. This may suggest the continued growth of Eu_2_Ti_2_O_7_ and/or Eu_2_O_3_ nanocrystallites. Unlike the columnar microstructures present at *x* < 50, the *x* = 0.77 film exhibited a fine-grained, smooth microstructure without distinct features (Fig. 3d). Consistent with previous observations, all films with *x* = 0.65–0.86 demonstrated red luminescence under 365 nm excitation (Fig. 3d). However, films with *x* = 0.80–0.86 became brittle and delaminated from the substrate following annealing.

The XRD pattern of an annealed film with *x* = 1 (*i.e.* Eu_2_O_3_) film exhibits characteristic peaks that indicate the formation of crystalline clusters (Fig. 3d), which can be associated with the monoclinic phase lattice (m-Eu_2_O_3_) (space group: *C*2/*m*, 12). The monoclinic lattice constants are determined to be *a* = 1.4108 nm, *b* = 0.3604 nm, and *c* = 0.8806 nm, with a corresponding crystallite size of 61 nm (eqn (SI 2)†).^32^ The additional broad peak observed at 2*θ* of ∼20° could be attributed to the quartz substrates (Fig. SI 9†).

## 4 Discussion

### 4.1 Spatial variation in Eu/(Eu + Ti)

Our finding shows that the combinatorial co-sputtering of TiO_2_ and Eu_2_O_3_ produces a variation in the *x* = Eu/(Eu + Ti) ratio as a function of sample positioning, which can be approximated by a logistic regression function. The profile is influenced by the deposition parameters, such as the positioning of the samples relative to each target, the distance between the targets, and the power density of each sputtering target. In this study, we demonstrated that the positioning of the samples plays a significant role in the variation of film compositions, where samples closer to the TiO_2_ target have lower *x* while those nearer to the Eu_2_O_3_ target have higher *x*.

The deposition process is influenced by both the sputtering yield and the sputtering flux, where the proximity of the targets plays a critical role. A larger distance between the targets and/or a lower power density of the sputtering targets may lead to a logistic regression profile with a gentler transition. Moreover, when targets are spaced too far apart, minimal to no overlap in sputtering flux occurs, leading to limited or no compositional variation in the deposited coatings. Conversely, a smaller distance between the sputtering targets and/or the application of higher power density to the targets results has a greater overlap of sputtering fluxes can be characterised as having a narrower compositional range and a logistic regression curve with a steeper slope. Equally, when the substrate–target distance is significantly increased, the area of the sputtering flux increases which also may lead to a larger mixed zone. Moreover, the results of the elemental analysis indicate a large variation of *x* from 0.18 to 0.78 in just a span of 55 mm takes place somewhere between the two magnetrons. While this is not explored in this study, we believe the logistic profile can be further tuned by varying the placement of the magnetrons and the power setting of each magnetron, thus opening a pathway to tailor film composition for rapid investigation or discovery of complex materials systems beyond our ETO model systems.

### 4.2 Effect of compositions and annealing on crystallography

As shown by the XRD pattern of as-deposited TiO_2_ (*x* = 0) thin films, the low surface free energy of anatase TiO_2_ facilitates the formation of long-range order,^46^ leading to the formation of the anatase phase of TiO_2_ during the magnetron sputtering deposition process. With an increase of *x* to 0.06, the XRD patterns of as-deposited ETO thin films (Fig. 3a) exhibit broad peaks, indicating that these films are dominated by an amorphous matrix, with anatase TiO_2_ nanocrystallites either absent or present in minimal amounts with diameters of <2 nm.^47^ This suggests that the incorporation of a small amount of Eu^3+^ disrupts the TiO_2_ long-range order and suppresses the formation of anatase TiO_2_ crystallites, rendering the films amorphous.^46^ Magnetron sputtering deposition may not provide sufficient thermal energy to overcome such energy barriers introduced by the long-range order disruption caused by Eu^3+^. Therefore, annealing is required to reorganise the films into a more stable and ordered lattice configuration.^48^ This contrasts with the previously reported studies that reported the formation of rutile TiO_2_ by magnetron sputtering or the presence of cubic phases of Eu_2_O_3_ without annealing.^28^

For as-deposited ETO films with *x* = 0.06, the EDS elemental mapping shows an O-deficient zone at the base and middle section of the film, while Eu and Ti are distributed uniformly throughout the entire thickness of the film. This suggests the formation of oxygen vacancies through substitution of Ti^4+^ by Eu^3+^
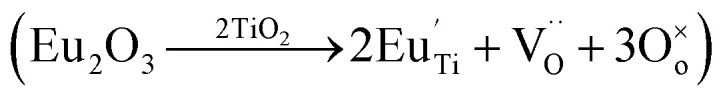
 to maintain the charge neutrality of the ETO films.^49,50^ A peak broadening and shifts of the E_g_ mode at ∼144 cm^−1^ in the Raman spectra further indicate structural distortions in the TiO_2_ lattice from oxygen vacancies (Fig. SI 12 and 13†). Note that the Raman spectra of these films only exhibit peaks associated with the Raman active modes of TiO_2_ at ∼401 cm^−1^ (B_1g_), ∼521 cm^−1^ (A_1g_ + B_1g_), and 637 cm^−1^ (E_g_), and are free from any peaks associated with the Raman active modes of Eu_2_Ti_2_O_7_ or Eu_2_O_3_. It is therefore essential to highlight that in this composition, there is no evidence of the formation of separate phases such as Eu_2_Ti_2_O_7_ or Eu_2_O_3_. This further suggests that the substitution of Ti^4+^ by Eu^3+^ takes place within the amorphous TiO_2_ matrix rather than phase separation into Eu_2_Ti_2_O_7_ or Eu_2_O_3_.

Upon annealing at 600 °C, crystallisation occurs in ETO thin films with *x* < 0.1, forming crystallite clusters of anatase and rutile phases of TiO_2_ (Fig. 3b).^51,52^ Although the onset of the anatase-to-rutile transition begins at 600 °C, prolonged annealing is generally required due to its slow kinetics. The presence of oxygen vacancies accelerate this transition by relaxing the oxygen sublattice, allowing the formation of rutile TiO_2_ at lower temperatures.^46^ Annealing provides the requisite thermal activation energy to enhance atomic mobility and overcome the energy barriers introduced by the long-range order disruption caused by Eu^3+^, thereby enabling the transition from an amorphous to a partially crystalline structure.^53^ The process commences with the nucleation of small crystalline clusters within the amorphous matrix, which subsequently expand as additional atoms align with the forming lattice structure.^54^

Note that the formation of anatase and rutile TiO_2_ by annealing does not lead to elemental segregation, as demonstrated by the unaltered spatial co-location of Eu^3+^ and Ti^4+^ within the submicron sensitivity limit of EDS analysis (Fig. 3a). In addition, the broadening of the characteristic TiO_2_ Raman peak at 144 cm^−1^ and the additional minor peaks between 200 and 400 cm^−1^ implies the strain and disruption of the TiO_2_ symmetry by the presence of lattice impurities or dopants (Fig. SI 12 and 13†).^55–57^ Together with the XRD analysis that indicates the absence of phase separation from the formation of Eu_2_Ti_2_O_7_ or Eu_2_O_3_ phases, this further suggests the high likelihood that some Eu^3+^ ions are incorporated into the anatase or rutile TiO_2_ clusters formed during annealing. To further confirm the formation of such clusters and the diffusion of Eu^3+^ into them as a substitutional of Ti^4+^, higher-resolution imaging and crystallographic techniques, such as transmission electron microscopy (TEM) with selective area electron diffraction (SAED), should be conducted in future studies.

At intermediate *x* (0.1 < *x* < 0.8), the anatase and rutile clusters progressively diminish as *x* increases. Notably, anatase and rutile phases are absent in the XRD patterns of ETO films with *x* > 0.26, indicating their complete suppression by high concentrations of Eu^3+^.^32^ At *x* = 0.26, the Eu^3+^ concentration within the TiO_2_ matrix reaches a threshold where cubic Eu_2_Ti_2_O_7_ forms, although primarily as nanocrystallites, since the annealing temperature may not be sufficiently high to overcome the energy barrier for long-range ordering.

At high *x* (*x* > 0.9), the opposite occurs, where Ti^4+^ may diffuse into the monoclinic Eu_2_O_3_ matrix. This is further implied by the similarity in the Raman spectra of annealed ETO with *x* = 0.98 to that of Eu_2_O_3_ thin films (*x* = 1). The Raman spectra for both films show several distinct peaks characteristic of the monoclinic Eu_2_O_3_ (Fig. SI 13†), indicating a transition from the amorphous to a more crystalline phase following the annealing process. Various peaks illustrated in the Raman spectra are 111 cm^−1^ (B_g_), 175 cm^−1^ (A_g_), 243 cm^−1^ (A_g_), 282 cm^−1^ (B_g_), 424 cm^−1^ (B_g_) and 583 cm^−1^ (E_g_), corresponding to the Raman active mode. Due to the limited thermal energy of the magnetron deposition process, long-range order does not fully develop, and annealing induces direct crystallisation into the monoclinic phase rather than transitioning through the cubic phase, which typically occurs above 1000 °C.^32^

### 4.3 Effect of compositions on photoluminescence properties

**Fig. 4:**
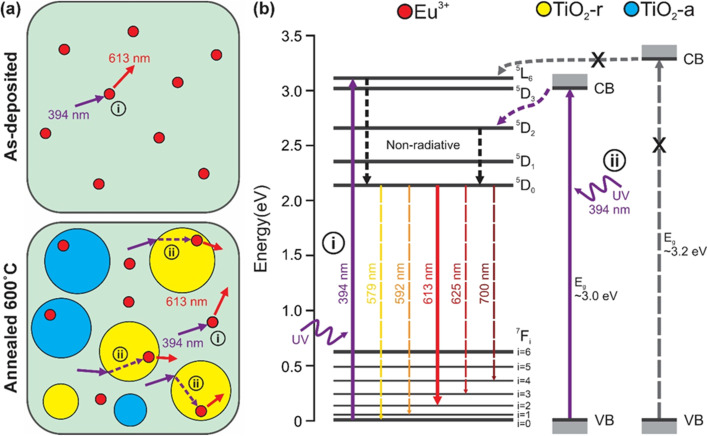
Schematic of (a) microstructures and main PL processes, (b) energy level, bandgap, and energy transfer diagram of Eu^3+^ ion, anatase TiO_2_ (TiO_2_-a), and rutile TiO_2_ (TiO_2_-r). Scenario (i) Eu^3+^ ions are excited by 394 nm UV light to the ^5^L_6_ level and generate non-radiative energy transfer to ^5^D_0_ levels, and radiative emission transitions originating from the ^5^D_0_ levels to ^7^F_*J*_ (*J* = 0, 1, 2, 3, 4) levels and the dominant emission wavelength is at 613 nm. Scenario (ii) the rutile phase of TiO_2_ with a bandgap of ∼3.0 eV is excited by 394 nm UV light. Energy transfer from TiO_2_ (TiO_2_-r) to Eu^3+^ ions occurs *via* non-radiative processes. Photon absorption and subsequent energy transfer by anatase TiO_2_ (TiO_2_-a) at this wavelength are largely inhibited due to its wider bandgap of ∼3.2 eV.

Our PL and XRD data indicate that as-deposited ETO films with *x* = 0.04–0.43 are mildly luminescent despite being amorphous. At *x* = 0.06, nearly all Eu^3+^ ions reside within the amorphous TiO_2_ matrix, which can be excited through the charge transfer band (CTB) of O^2−^ → Eu^3+^ (280 nm) or directly through ^7^F_0_ → ^5^L_6_ (394 nm) and ^7^F_0_ → ^5^D_2_ (465 nm) transitions of Eu^3+^ itself (Fig. SI 20†). CTB generally has a wider absorption cross-section that leads to a more intense PL emission.^58–62^ Direct excitation of ^7^F_0_ → ^5^L_6_ and ^7^F_0_ → ^5^D_2_ are Laporte-forbidden transitions that lead to a narrow absorption cross-section and a weak PL emission.^18^ This direct excitation does not require energy transfer from TiO_2_ and, hence, is independent of the TiO_2_ bandgap (Fig. 4ai). The bandgap of ETO thin films remains largely unaffected by the annealing process (Fig. SI 17 and 18†).

As indicated by EDS mapping, Eu^3+^ ions are found to be well dispersed within the amorphous TiO_2_ matrix at *x* = 0.06. This mitigates the close proximity of Eu^3+^ ions, thereby reducing concentration quenching from energy migration among Eu^3+^ ions and leading to stronger PL emission.^18^ The presence of oxygen vacancies lowers the local Madelung potential and effectively reduces the energy required for CTB excitation, improving Eu^3+^ excitation efficiency and further enhancing PL emission.^24^ However, here we found that CTB is weaker than the direct ^7^F_0_ → ^5^L_6_ excitation. This suggests that the PL emission of Eu^3+^ in an amorphous TiO_2_ matrix suffers from rapid energy dissipation through non-radiative pathways, such as defect-mediated quenching from excessive oxygen vacancies or hydroxyl groups and strong phonon interactions with the surrounding matrix.^58–60^ While we do not find a direct evidence of hydroxyl groups, their presence in amorphous TiO_2_ from adsorption of moisture 
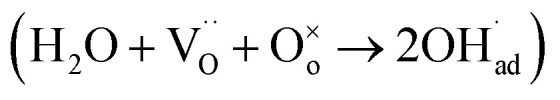
, particularly on magnetron sputtered TiO_2_ thin films, has been previously reported.^18,38,63,64^

After initial excitation, the electrons relax to the ^5^D_0_ band edge through non-radiative cascading single/multi-phonon relaxation from ^5^L_6_.^18^ This is followed by the distinctive radiative transition in the visible spectral range from ^5^D_0_ to ^7^F_*J*_, with *J* = 0, 1, 2, 3, and 4 (Fig. 4bi).^26^ According to Judd-Ofelt theory, electric-dipole (ED) transitions from the ^5^D_0_ state to odd *J* levels are forbidden. Therefore, the primary ED transitions expected are ^5^D_0_ → ^7^F_*J*_ with *J* = 2, 4, and 6.^32^ Among all radiative transitions, we observed that the most intense and well-defined red emission peak is at 613 nm, which corresponds to the ED allowed transition of ^5^D_0_ → ^7^F_2_.^33^ This transition is often referred to as the “hypersensitive” transition due to high responsiveness of its intensity to the local environment of Eu^3+^.^65^ The transitions ^5^D_0_ → ^7^F_*J*_ with *J* = 0, 3, and 5, which are associated to forbidden odd *J* levels, exhibit low intensity. However, the ^5^D_0_ → ^7^F_1_ transition at 592 nm is an exception, as it arises from a magnetic-dipole (MD) transition, with its intensity governed by the spin–orbit coupling of the 5D and 7F multiplets.^32^

Note that the PL emission intensity from ^5^D_0_ → ^7^F_2_ transition reaches a maximum at *x* = 0.08. This is in agreement with the previous study where *x* = 0.07–0.1 showed the optimum PL emission intensity, as it has the most available Eu^3+^ emitting centres in the amorphous TiO_2_ matrix.^18,29^ A further increase in *x* leads to a rapid decrease in PL emission intensity due to concentration quenching, where a smaller distance between adjacent Eu^3+^ leads to non-radiative energy migration between these ions.^18,66^ For *x* > 0.44, a strong concentration quenching from excessive energy migration among Eu^3+^ ions results in negligible PL emission intensity. It is also worth noting that the PL emission intensity from the ^5^D_0_ → ^7^F_4_ transition reaches a maximum at *x* = 0.26. As indicated by XRD analysis, this transition can be attributed to the presence of the Eu_2_Ti_2_O_7_ phase. Anomalously high emission intensities of the ^5^D_0_ → ^7^F_4_ transition have been observed when Eu^3+^ ions occupy sites with D_4d_ symmetry.^67^

### 4.4 Effect of annealing on photoluminescence properties

Following annealing at 600 °C, clusters of anatase and rutile phases form within the amorphous TiO_2_ matrix. The growth of these crystalline clusters during annealing enables a portion of the Eu^3+^ ions to diffuse into these clusters. This allows ETO films with *x* = 0.06–0.18 to exhibit significantly higher excitation efficiency due to energy transfer from the crystalline TiO_2_ to the Eu^3+^ ions. Although the correlation between crystallite size and PL emission intensity is not fully explored in this study, larger crystalline TiO_2_ clusters may lead to a stronger PL emission from lower defect-mediated and concentration quenching as well as an increased likelihood in energy transfer from crystalline TiO_2_ to Eu^3+^ and O^2−^ → Eu^3+^ CTB.^18,29,62,66^ However, excessively large TiO_2_ cluster may weaken PL emission from non-radiative recombination by TiO_2_ prior to energy transfer to Eu^3+^ and phase separation in a form of Eu_2_Ti_2_O_7_ or Eu_2_O_3_.^68^

Note that annealing at a lower temperature of 450 °C, which is sufficient for the growth of anatase crystallites but well below the onset of the anatase-to-rutile transition, results in a negligible change in PL emission intensity (Fig. SI 14 and 15†).^69^ This suggests that anatase TiO_2_ is ineffective at absorbing 394 nm photons. Anatase TiO_2_ possesses a wider bandgap at ∼3.2 eV compared to its rutile counterpart at ∼3.0 eV,^38^ and such difference is critical in determining their photophysical behaviour. The presence of rutile TiO_2_ crystallites enables more effective absorption of photons at 394 nm, as they are more likely to be excited than the anatase crystallites (Fig. 4aii). Consequently, absorption by anatase TiO_2_ is not expected to occur (Fig. 4bii). While absorption by rutile TiO_2_ does not eliminate the direct ^7^F_0_ → ^5^L_6_ excitation of Eu^3+^, it facilitates energy transfer to the ^5^D_2_ of Eu^3+^, which then relaxes to the ^5^D_0_ band edge through non-radiative cascading single/multi-phonon relaxation.^18^ This is followed by radiative transitions from ^5^D_0_ to ^7^F_*J*_ with the transition of ^5^D_0_ → ^7^F_2_ at 613 nm being the most intense and well-defined red emission observable in the visible spectral range (Fig. 4bii).

The presence of oxygen vacancies may introduce energy levels 0.75–1.18 eV below the conduction band and create localised trap states in the TiO_2_ matrix.^24^ While these trap states may hinder energy transfer from TiO_2_ to Eu^3+^, they may also effectively reduce the energy required for electronic transitions, allowing sub-bandgap excitation to promote electrons from these defect states into the conduction band or from the valence band to these intermediate states.^70^ This mechanism may facilitate energy transfer from anatase TiO_2_ to Eu^3+^ even with sub-bandgap excitation. However, the removal of the O-deficient zone at the base of the film suggests that annealing heals oxygen vacancies through the chemisorption of O into TiO_2_
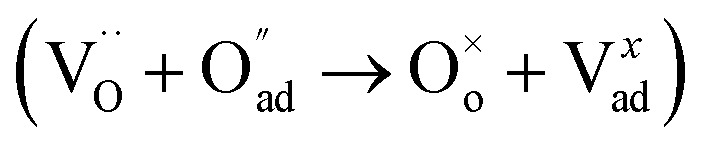
.^64^ This improves energy transfer from TiO_2_ to Eu^3+^ and reduces the non-radiative pathways from defect-mediated quenching, leading to a stronger PL emission.

At higher incorporation of Eu^3+^, such as at *x* = 0.26 and *x* = 0.77 (Fig. 3), crystalline TiO_2_ phases disappear along with the formation of nanocrystalline cubic Eu_2_Ti_2_O_7_ phase. This results in reduced photon absorption and diminished PL intensity (Fig. 4ai). While ETO films with *x* = 0.26 and *x* = 0.77 lack long-range order after annealing at 600 °C, larger crystallites may form if the annealing temperature is further raised to 1000 °C.^71,72^ For *x* = 1, the formation of monoclinic Eu_2_O_3_ crystallites limits the photon absorption to Laporte-forbidden ^7^F_0_ → ^5^L_6_ and ^7^F_0_ → ^5^D_2_ transitions with a narrow absorption cross-section (Fig. 4bi).^18^ A strong concentration quenching further weaken the PL emission of Eu_2_O_3_ thin films.^73–75^

The emission peaks observed at 613 nm and 625 nm for *x* = 0.78 and *x* = 0.98 are attributed to the Stark-splitting of the ^5^D_0_ → ^7^F_2_ transition, which is influenced by the local site symmetry of the Eu^3+^ ions.^76^ The presence of two distinct peaks at these wavelengths suggests that the local structure surrounding the Eu^3+^ ions exhibits low symmetry, such as orthorhombic, triclinic, or monoclinic configurations.^77^ XRD analysis for *x* = 0.78 and *x* = 0.98 confirms that Eu_2_O_3_ has a monoclinic structure. The low site symmetry associated with monoclinic Eu_2_O_3_ likely contributes to enhanced Stark-splitting, resulting in the dominant peak at 625 nm for *x* = 0.98. In contrast, for *x* = 0.26, Eu_2_Ti_2_O_7_ exhibits a cubic structure with high site symmetry. This higher symmetry leads to significantly weaker Stark-splitting, with the primary peak for the ^5^D_0_ → ^7^F_2_ transition observed at 613 nm.^78^

## 5 Conclusions and outlook

In this study, we successfully demonstrate the advantages of combinatorial sputtering for the rapid fabrication of materials with diverse characteristics through minimal experimental iterations. This approach enables the fabrication of an ETO film with varying *x* = Eu/(Eu + Ti) ranging from *x* = 0 to *x* = 1 in a single deposition process. The maximum PL emission intensity at 613 nm, attributed to the ^5^D_0_ → ^7^F_2_ transition, occurs at *x* = 0.08 for both as-deposited films and those annealed at 600 °C. For films with *x* < 0.50, the as-deposited films exhibit a vertically oriented columnar microstructure with observable PL characteristics despite their amorphous crystallography with no discernible peaks in XRD. After annealing, these films form clusters of nano-sized anatase and rutile TiO_2_ crystallites, into which Eu^3+^ ions diffuse. This leads to an energy transfer from TiO_2_ to Eu^3+^ that significantly enhances the PL emission intensities.

For future work, addressing the spatial resolution limitations of XRD, such as by employing micro XRD, will be crucial to accurately establish the relationship between film crystallography, PL mechanisms, and intensity. Future studies should also consider the influence of other film characteristics, such as thickness, residual stress, and surface morphology, as well as environmental factors including humidity and oxygen exposure, on the PL properties of the film. As demonstrated herein, ETO thin films can be deposited on PDMS substrates and maintain structural integrity and PL properties, even when subjected to mechanical deformation. The temporal stability of the PL properties of ETO films in air, under ambient or more demanding conditions, warrants further investigation to establish confidence in their long-term usability. This highlights the potential for flexible ceramic luminescent thin films and informs further investigation into their advantages over flexible polymeric luminescent films that often rely on dye molecules or nanocrystalline particulates as luminophores. While the findings presented herein are focused on demonstrating the effect of composition and annealing on crystallography and PL properties of ETO films, they provide a foundation for the rational development of luminescent thin films, essential for wide applications ranging from solar photovoltaics to electronic displays. The combinatorial sputtering approach employed herein can be expanded to systematically explore other model systems that critically depend on the composition of the constituent elements.

## Author contributions

Junfeng Chen: conceptualisation, validation, investigation, methodology, formal analysis, writing – original draft, visualisation, data curation, writing – review & editing. Jeff Rao: supervision, writing – review & editing. Adrianus Indrat Aria: conceptualisation, supervision, visualisation, funding acquisition, writing – original draft, writing – review & editing.

## Conflicts of interest

There are no conflicts to declare.

## Supplementary Material

RA-015-D5RA04076K-s001

## Data Availability

Data supporting this study are openly available from Cranfield University Research Data (CORD) at https://doi.org/10.57996/cran.ceres-2684.
