# Supplementary material for: Combinatorial sputtering of photoluminescent europium titanium oxide thin films
Source: RSC Adv. 2025 Aug 1;15(33):27415–28. doi: 10.1039/d5ra04076k (PMC12314876; doi:10.1039/d5ra04076k)
Supplement: RA-015-D5RA04076K-s001 [file RA-015-D5RA04076K-s001.pdf]

Supplementary Information (SI) for RSC Advances.
This journal is © The Royal Society of Chemistry 2025

Supporting Information

1 Equations

1.1 Logistic Regression

The compositional relationship between x = Eu/(Eu+Ti) and the position L (in mm) from the left edge of the wafer can be approximated by the following logistic regression (Equation 1):

$$x(L) = \frac{1}{1 + e^{-k(L - L_0)}} \quad (1)$$

with L_0 is the inflection position (84.66 ± 1.59 mm) and k is the curve steepness (0.048 ± 0.003).

1.2 Debye-Scherrer

The Debye-Scherrer equation is applied to determine the average crystal grain size.

$$D = \frac{k\lambda}{\beta \cos\theta} \quad (2)$$

D is the crystallite size in nm, *k* is the shape constant (0.9) (The shape factor typically holds a value around 0.9, although it can vary depending on the specific geometry of the crystallite), λ is the X-ray wavelength of Cu-kα radiation in nm, θ is the Bragg's angle in degrees, and β is the observed peak width at half-maximum peak height in rad.[1]

1.3 Tauc plot

The Tauc plot equation is exhibited as follows:

$$(\alpha h\upsilon)^{1/n} = A(h\upsilon - E_g) \quad (3)$$

$$(\alpha h\upsilon)^2 \ vs\ h\upsilon \quad (4)$$

In the context of this thesis, the symbol α represents the absorption coefficient, which is dependent on wavelength and expressed as $\alpha(\lambda)$. Other variables include *h*, representing Planck's constant; *Eg*, denoting the optical band gap of a semiconductor; υ, signifying frequency; *A*, representing a proportionality constant; and *n*, which refers to the Tauc exponent.

1.4 Lognormal distribution

The Lognormal distribution equation is applied to determine the PL emission intensity profile for the as-deposited films. σ is the shape parameter, μ is the location parameter, is exhibited as follows:

$$\mathrm{f}(x) = \frac{1}{\sigma x\sqrt{2\pi}} e^{\frac{-(\ln x - \mu)^2}{2\sigma^2}} \qquad (5)$$

1.5 Weibull distribution

The Weibull distribution equation is applied to determine the PL emission intensity profile for the annealed films. α is the shape parameter, β is the scale parameter, x is the location parameter, is exhibited as follows:

$$\mathrm{f}(x) = \frac{\alpha}{\beta^{\alpha}} x^{\alpha - 1} e^{-(\frac{x}{\beta})^{\alpha}} \qquad (6)$$

2 Microstructures, PL spectra and optical spectra

2.1 Film surface morphology

Figures 1–4 in the SI presented SEM images of ETO thin films at varying magnifications. The SEM images on the left were captured at 500× magnification, while those on the right were captured at 10,000× magnification. Scale bars were included for clarity: a 100 μm scale bar corresponds to the 500× magnification images, and a 5 μm scale bar corresponds to the 10,000× magnification images. Figures 1 and 2 in the SI depicted the as-deposited ETO thin films, whereas Figures 3&4 in the SI showed the ETO thin films after annealing at 600°C. These SEM images effectively reveal the surface microstructures of the ETO thin films, highlighting the compositional variations and the influence of annealing temperature on their morphology.

The surface microstructure of the as-deposited x = Eu/(Eu+Ti) thin films is captured in top view by SEM (Figure 1 & Figure 2). These SEM images were acquired with magnifications of 500x and 10,000x and an acceleration voltage of 10 keV. The surface morphology of thin films deposited near the TiO_2 target, with x below 0.5, reveals minute dense protrusions. These surface features can be attributed to the columnar microstructures, as confirmed by cross-sectional imaging. The thin films as-deposited with x at 0.05, 0.06, 0.08, 0.18, 0.25 and 0.44 were depicted in the SEM images (Figure 1). The thin films with x at 0.65, 0.76, 0.88, 0.96, 0.98 and 0.99 were depicted in the SEM images (Figure 2)

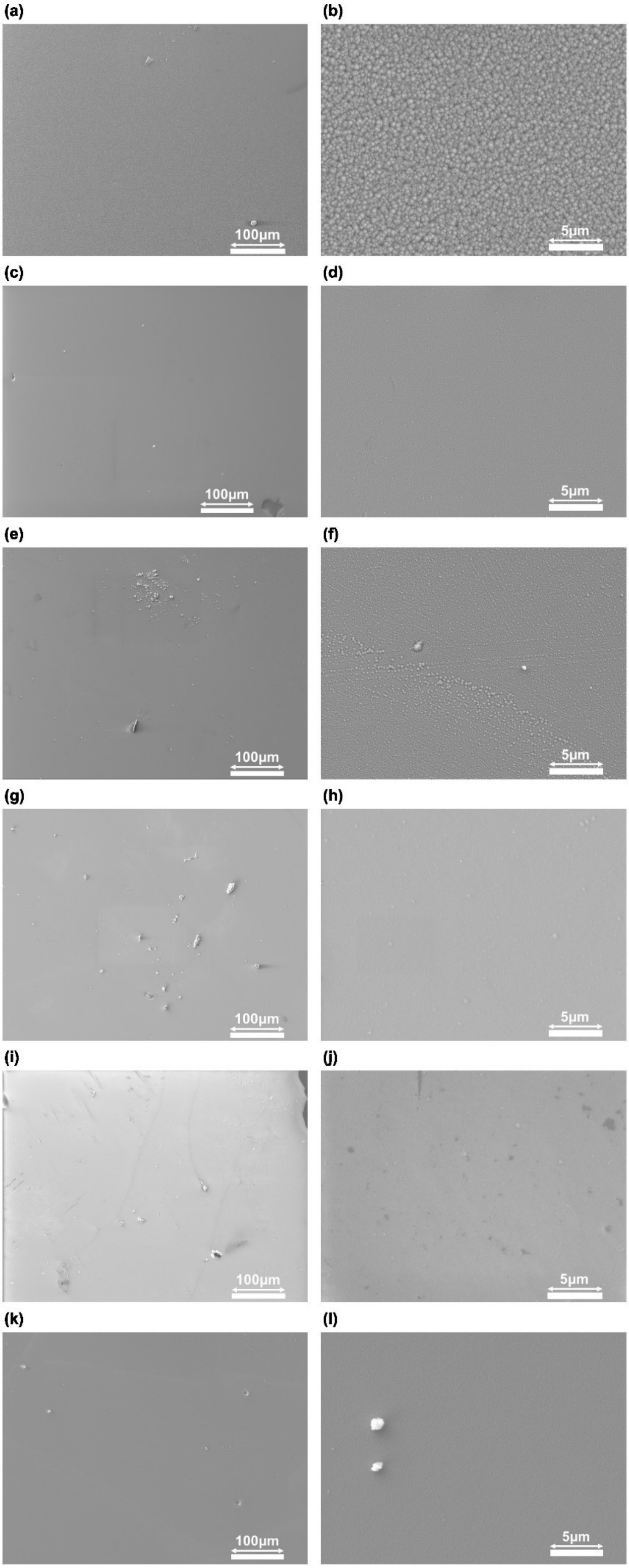
(a)
100μm
(b)
5μm
(c)
100μm
(d)
5μm
(e)
100μm
(f)
5μm
(g)
100μm
(h)
5μm
(i)
100μm
(j)
5μm
(k)
100μm
(l)
5μm

1

Figure 1 SEM images for the surface of the Eu-doped TiO_2 as-deposited with x at (a) & (b) 0.05, (c) & (d) 0.06, (e) & (f) 0.08, (g) & (h) 0.18, (i) & (j) 0.25 and (k) & (l) 0.44.

(a) (b) (c) (d) (e) (f) (g) (h) (i) (j) (k) (l)

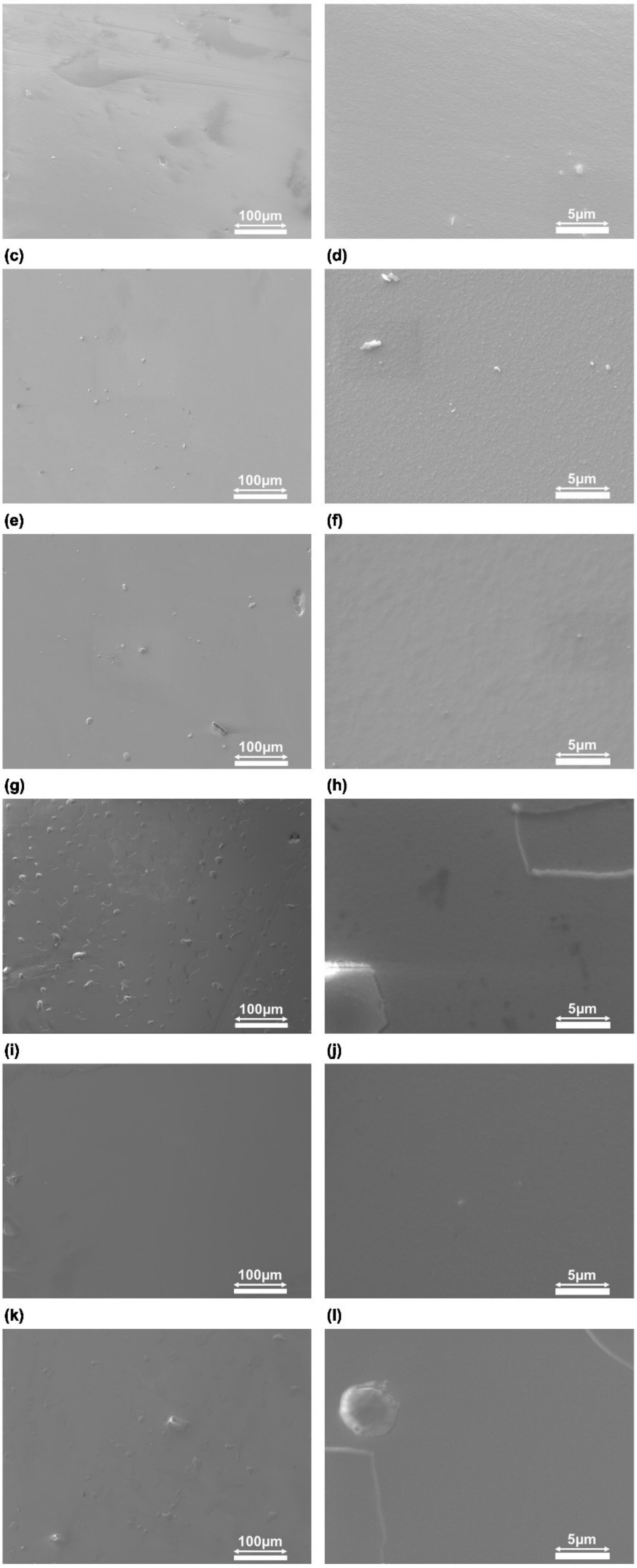

1

Figure 2 SEM images for the surface of the Eu-doped TiO_2 as-deposited with x at (a) & (b) 0.65, (c) & (d) 0.76, (e) & (f) 0.88, (g) & (h) 0.96, (i) & (j) 0.98 and (k) & (l) 0.99.

The surface microstructure of the Eu-doped TiO_2 thin films after annealing at 600˚C was examined in top view using SEM (Figure 3 & Figure 4). These SEM images were acquired with magnifications of 500x and 10,000x and an acceleration voltage of 10 keV. Subtle dense protrusions were observed on the surfaces of thin films deposited near the TiO_2 target with x below 0.5, which can be attributed to the columnar microstructures identified in cross-sectional imaging. After annealing at 600°C, these protrusions became more pronounced, reflecting changes in the microstructure. Additionally, annealing induced the formation of numerous cracks on several thin film samples. The thin films after annealing at 600˚C with x at 0.05, 0.06, 0.08, 0.18, 0.26 and 0.44 were depicted in the SEM images (Figure 3). The thin films after annealing at 600˚C with x at 0.65, 0.77, 0.88, 0.96, 0.98 and 0.99 were depicted in the SEM images (Figure 4).

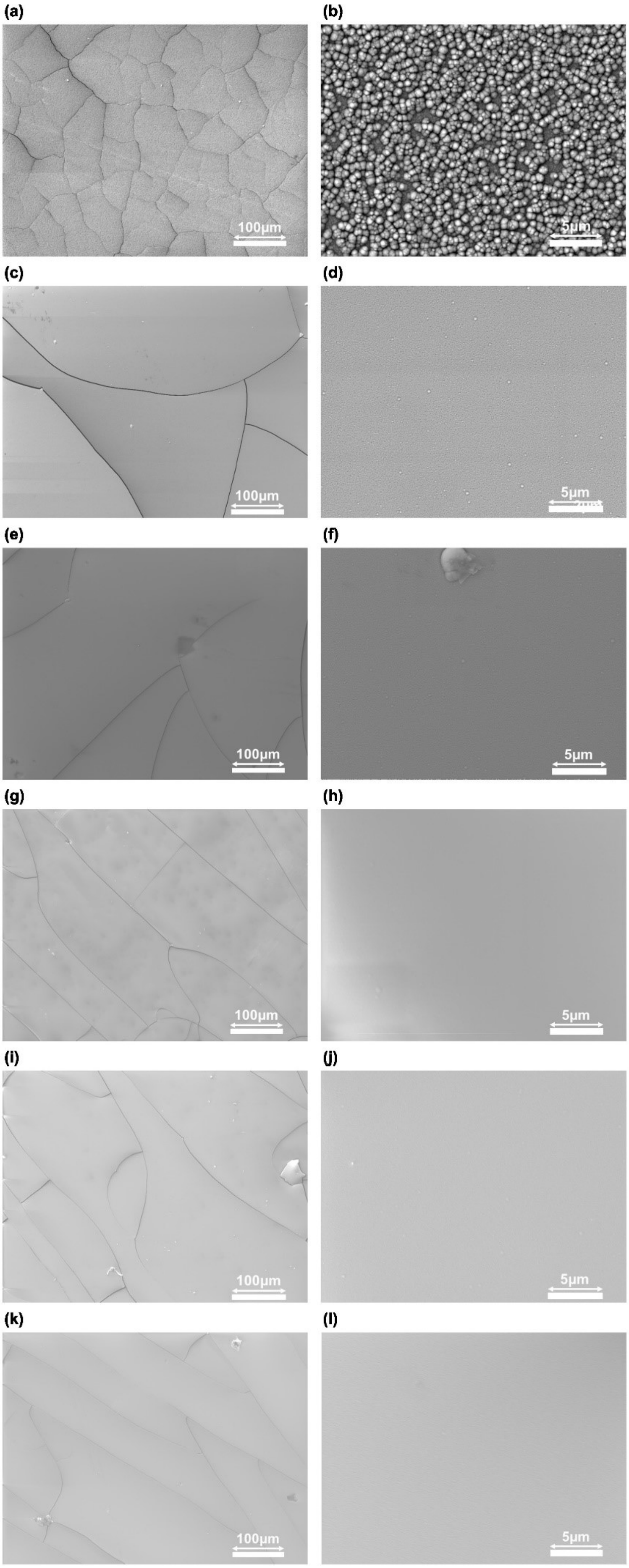
(a)
100μm
(b)
5μm
(c)
100μm
(d)
5μm
(e)
100μm
(f)
5μm
(g)
100μm
(h)
5μm
(i)
100μm
(j)
5μm
(k)
100μm
(l)
5μm

Figure 3 SEM images for the surface of the Eu-doped TiO2 after annealing at 600°C with x at (a) & (b) 0.05, (c) & (d) 0.06, (e) & (f) 0.08, (g) & (h) 0.18, (i) & (j) 0.26 and (k) & (l) 0.44.

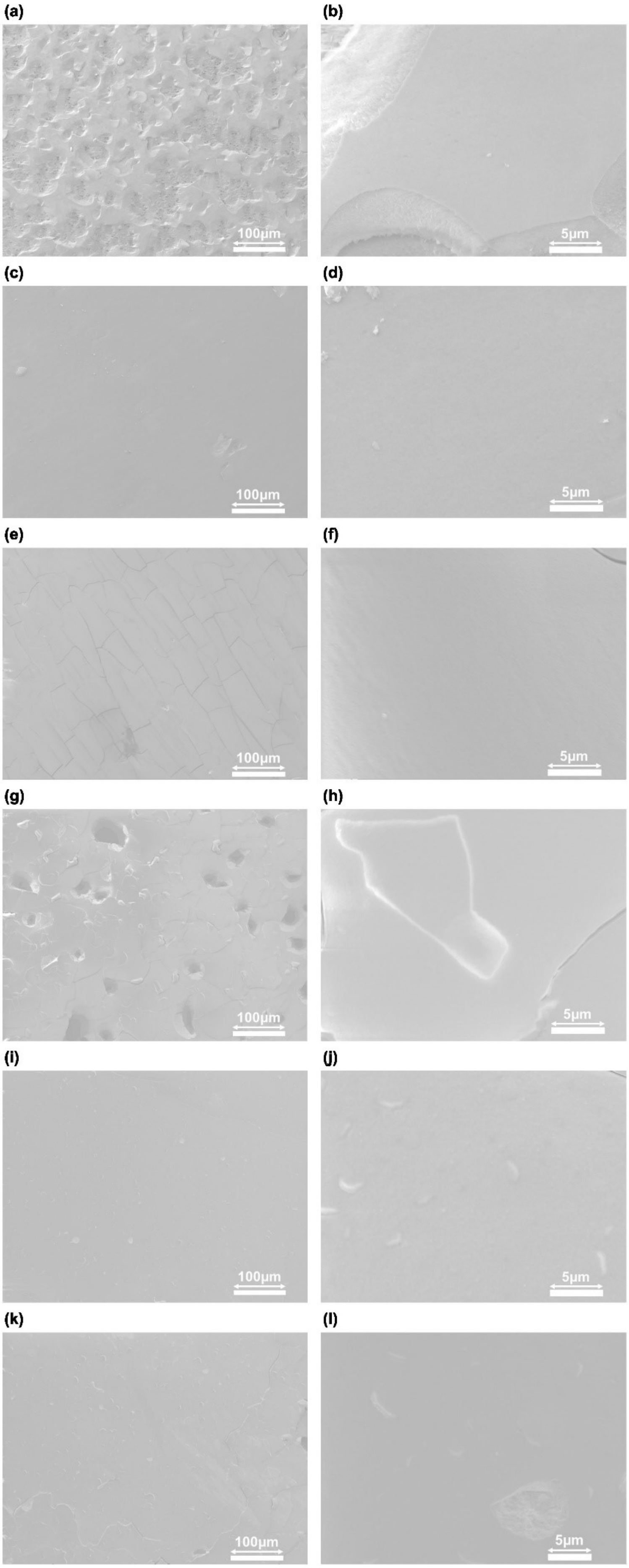

(a)
100μm
(b)
5μm
(c)
100μm
(d)
5μm
(e)
100μm
(f)
5μm
(g)
100μm
(h)
5μm
(i)
100μm
(j)
5μm
(k)
100μm
(l)
5μm

Figure 4 SEM images for the surface of the Eu-doped TiO_2 after annealing at 600°C with x at (a) & (b) 0.65, (c) & (d) 0.77, (e) & (f) 0.88, (g) & (h) 0.96, (i) & (j) 0.98 and (k) & (l) 0.99.

2.2 EDS mapping

Comprehensive analysis of the chemical composition of these thin films, including elemental mapping before and after annealing, is conducted using EDS analysis (Figure 5, Figure 6, Figure 7, and Figure 8). These EDS maps illustrate thin films featuring different x at 0.05, 0.06, 0.08, 0.18, 0.25, 0.44, 0.65, 0.76, 0.88, 0.96, 0.98, 0.99. The elemental analysis conducted via EDS mapping reveals that Eu, Ti, and O atoms maintain even distribution within the films both before and after annealing at 600˚C. The EDS maps display denser Ti atoms and sparser Eu atoms for thin films with lower Eu concentrations, associated with sputtering positions closer to the TiO_2 target. Conversely, EDS maps exhibit denser Eu atoms and sparser Ti atoms for thin films with higher Eu concentration, linked with sputtering positions nearer to the Eu_2O_3 target.

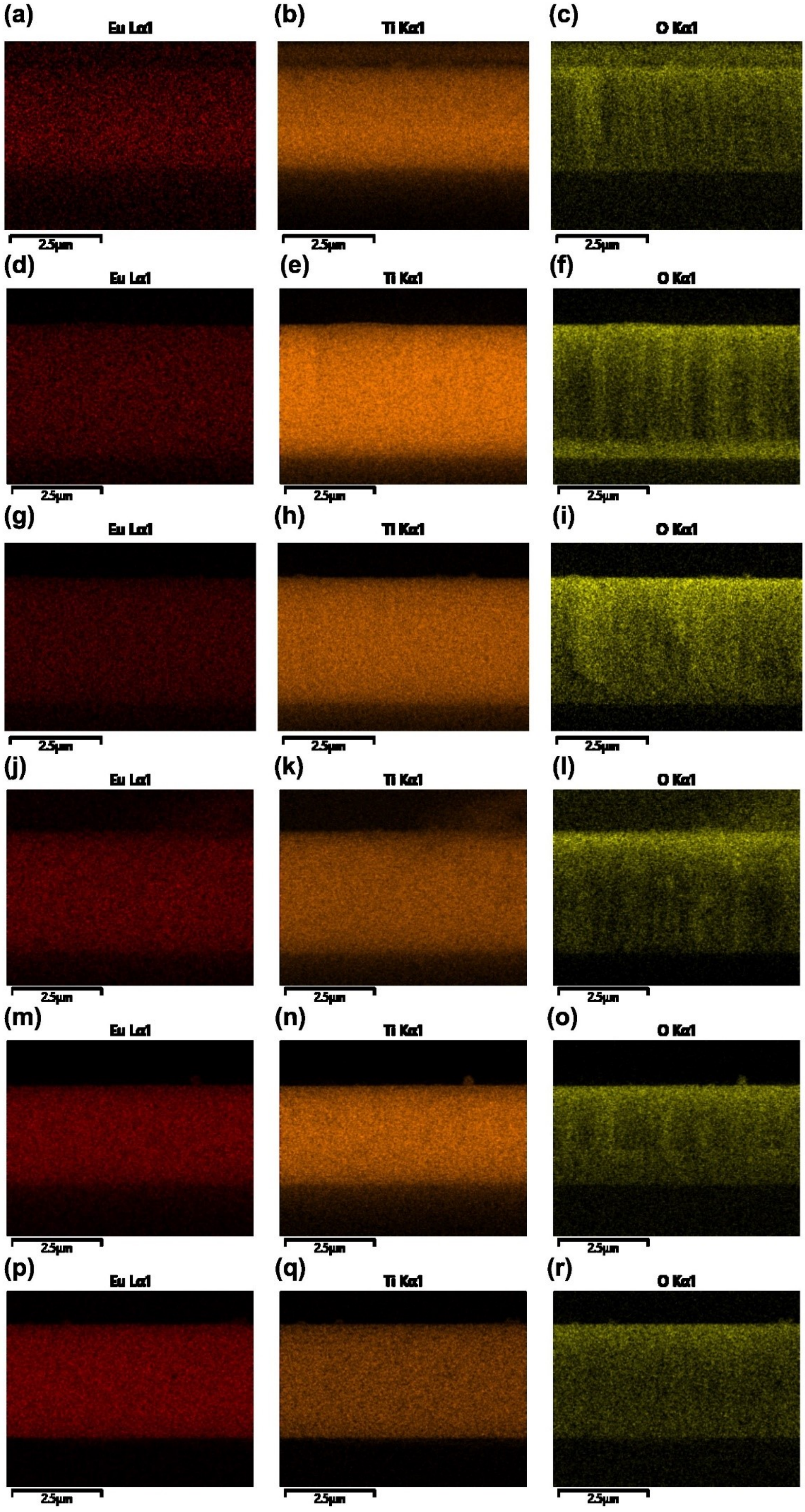
(a)
Eu Lα1
(b)
Ti Kα1
(c)
O Kα1
2.5μm
(d)
Eu Lα1
(e)
Ti Kα1
(f)
O Kα1
(g)
Eu Lα1
(h)
Ti Kα1
(i)
O Kα1
(j)
Eu Lα1
(k)
Ti Kα1
(l)
O Kα1
(m)
Eu Lα1
(n)
Ti Kα1
(o)
O Kα1
(p)
Eu Lα1
(q)
Ti Kα1
(r)
O Kα1

Figure 5 EDS mapping for the samples As-deposited with Eu, Ti and O composition in thin films at different Eu concentrations, x = (a) (b) (c) 0.05, (d) (e) (f) 0.06, (g) (h) (i) 0.08, (j) (k) (l) 0.18, (m) (n) (o) 0.25, and (p) (q) (r) 0.44.

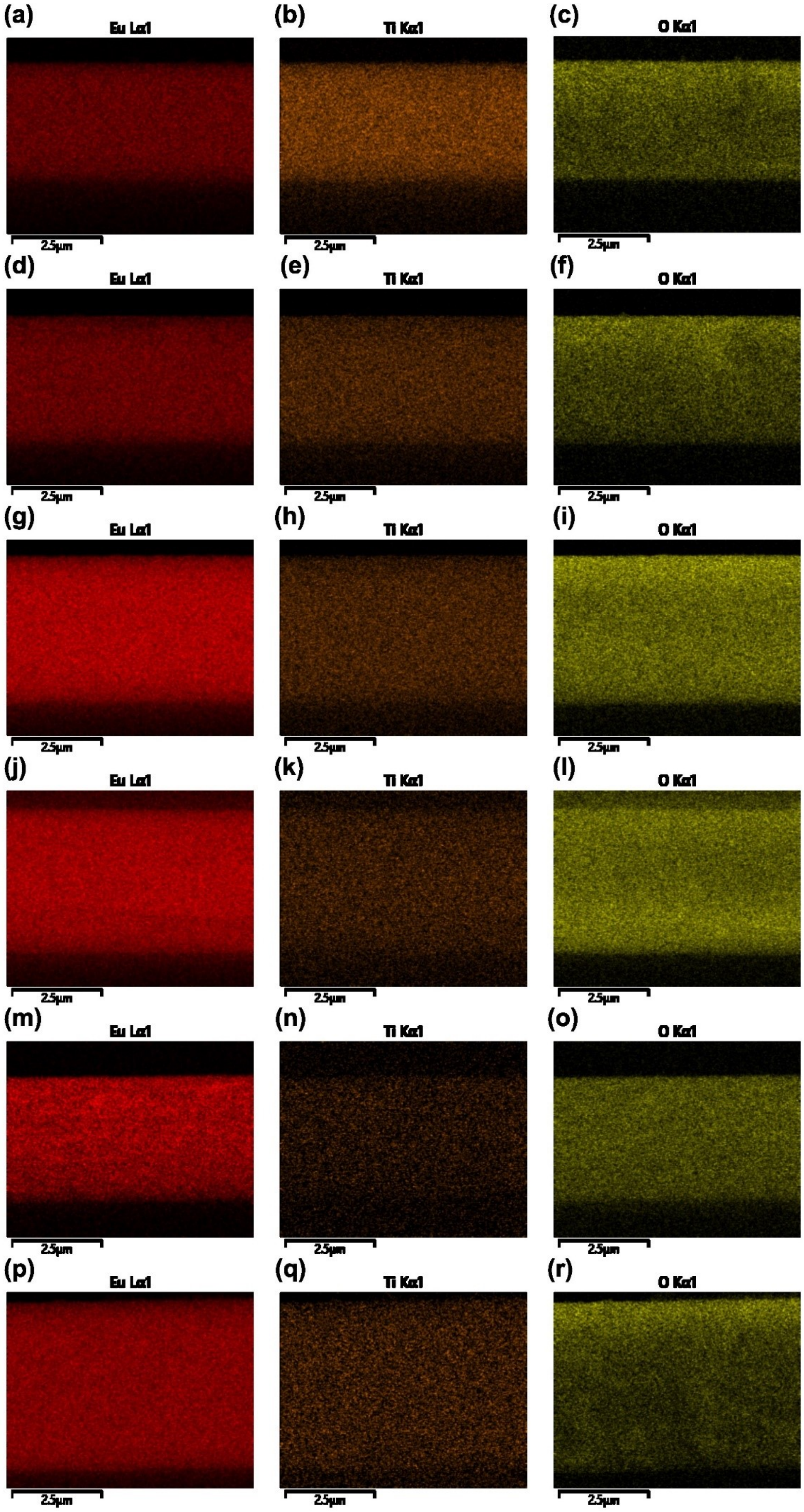
(a)
Eu Lα1
(b)
Ti Kα1
(c)
O Kα1
(d)
Eu Lα1
(e)
Ti Kα1
(f)
O Kα1
(g)
Eu Lα1
(h)
Ti Kα1
(i)
O Kα1
(j)
Eu Lα1
(k)
Ti Kα1
(l)
O Kα1
(m)
Eu Lα1
(n)
Ti Kα1
(o)
O Kα1
(p)
Eu Lα1
(q)
Ti Kα1
(r)
O Kα1
2.5µm

1

Figure 6 EDS mapping for the samples as-deposited with Eu, Ti and O composition in thin films at different Eu concentrations, x = (a) (b) (c) 0.65, (d) (e) (f) 0.75, (g) (h) (i) 0.88, (j) (k) (l) 0.96, (m) (n) (o) 0.98, (p) (q) (r) 0.99.

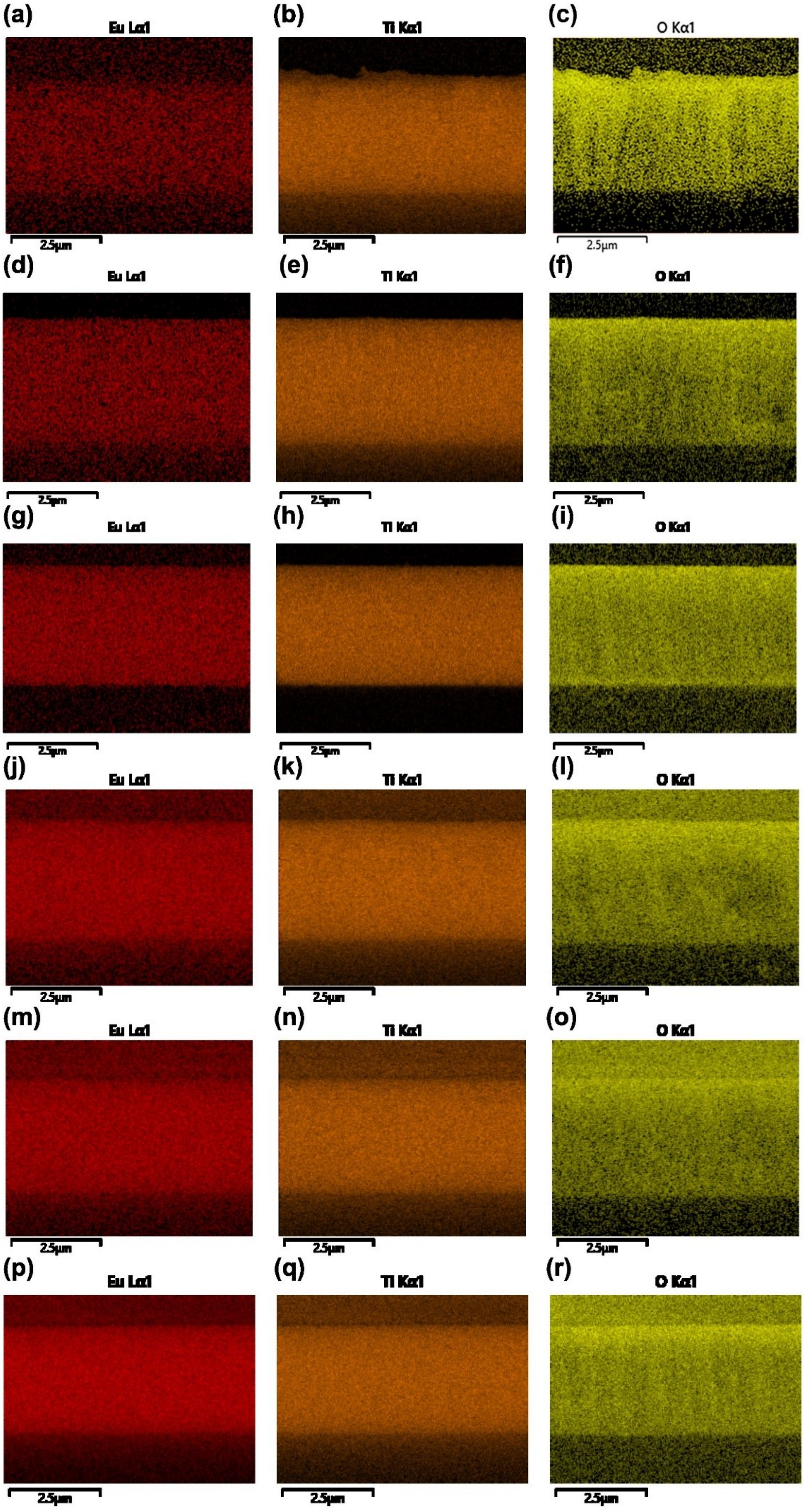
(a)
Eu Lα1
2.5μm
(b)
Ti Kα1
2.5μm
(c)
O Kα1
2.5μm
(d)
Eu Lα1
2.5μm
(e)
Ti Kα1
2.5μm
(f)
O Kα1
2.5μm
(g)
Eu Lα1
2.5μm
(h)
Ti Kα1
2.5μm
(i)
O Kα1
2.5μm
(j)
Eu Lα1
2.5μm
(k)
Ti Kα1
2.5μm
(l)
O Kα1
2.5μm
(m)
Eu Lα1
2.5μm
(n)
Ti Kα1
2.5μm
(o)
O Kα1
2.5μm
(p)
Eu Lα1
2.5μm
(q)
Ti Kα1
2.5μm
(r)
O Kα1
2.5μm

Figure 7 EDS mapping for the samples after annealing at 600˚C with Eu, Ti and O composition in thin films at different Eu concentrations, x = (a) (b) (c) 0.05, (d) (e) (f) 0.06, (g) (h) (i) 0.08, (j) (k) (l) 0.18, (m) (n) (o) 0.26, (p) (q) (r) 0.44.

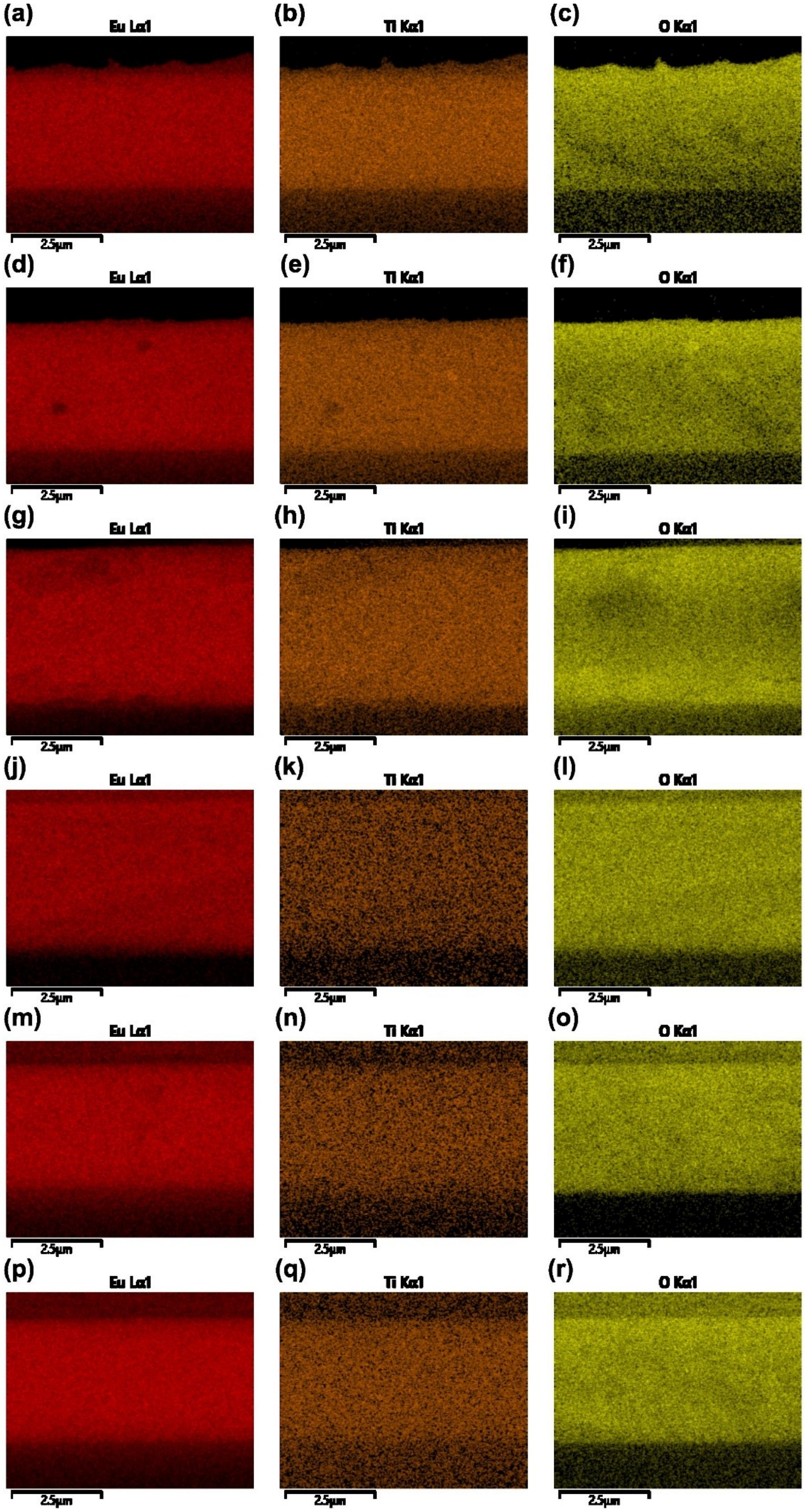

1

Figure 8 EDS mapping for the samples after annealing at 600˚C with Eu, Ti and O composition in thin films at different Eu concentrations, x = (a) (b) (c) 0.65, (d) (e) (f) 0.77, (g) (h) (i) 0.88, (j) (k) (l) 0.96, (m) (n) (o) 0.98, (p) (q) (r) 0.99.

2.3 XRD

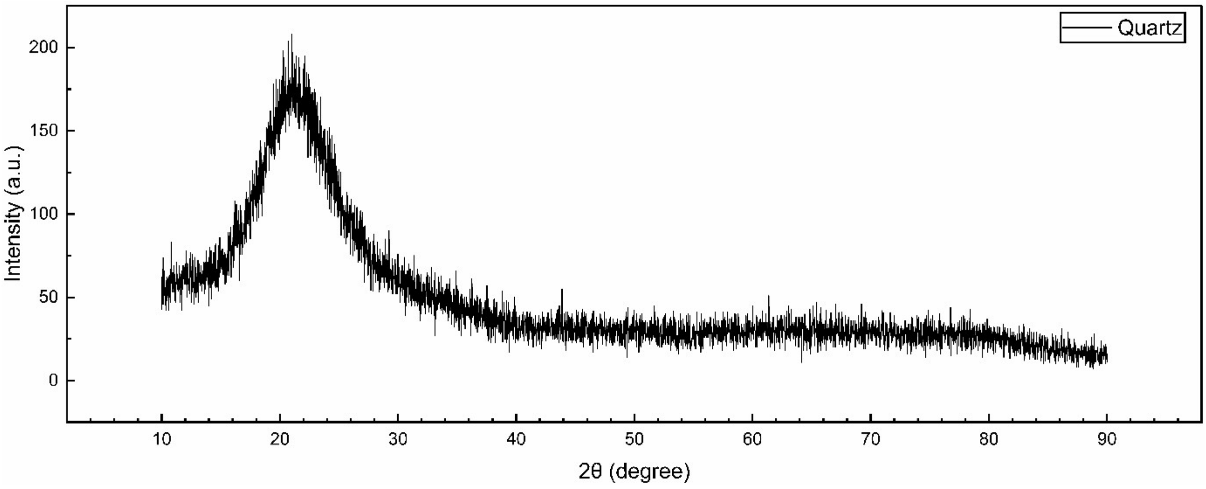

Figure 9 XRD patterns for the Quartz substrate from powder XRD.

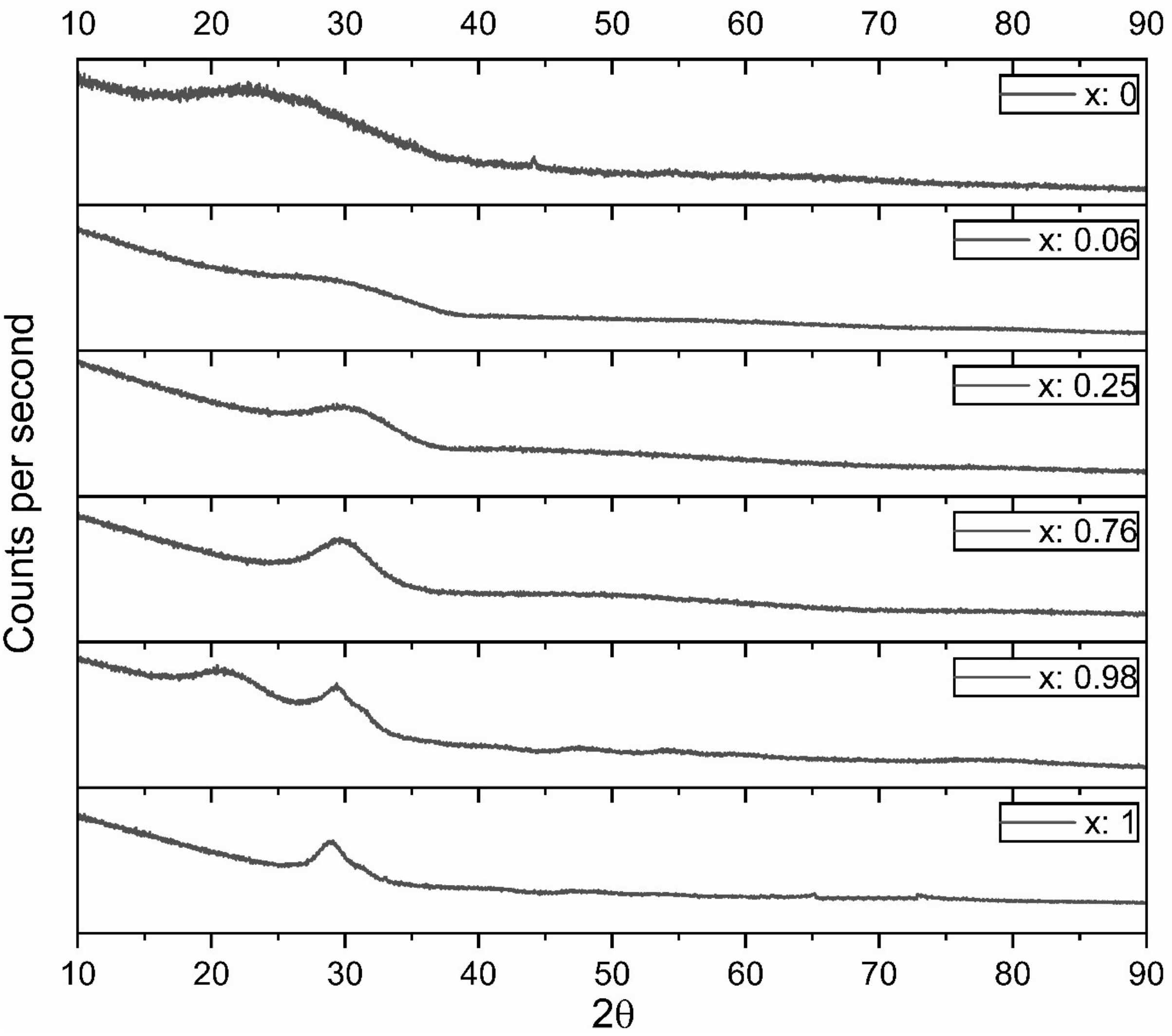

Figure 10 Thin film XRD patterns for the as-deposited ETO thin films with x = 0, 0.06, 0.25, 0.76, 0.98 and 1 Eu/(Eu+Ti).

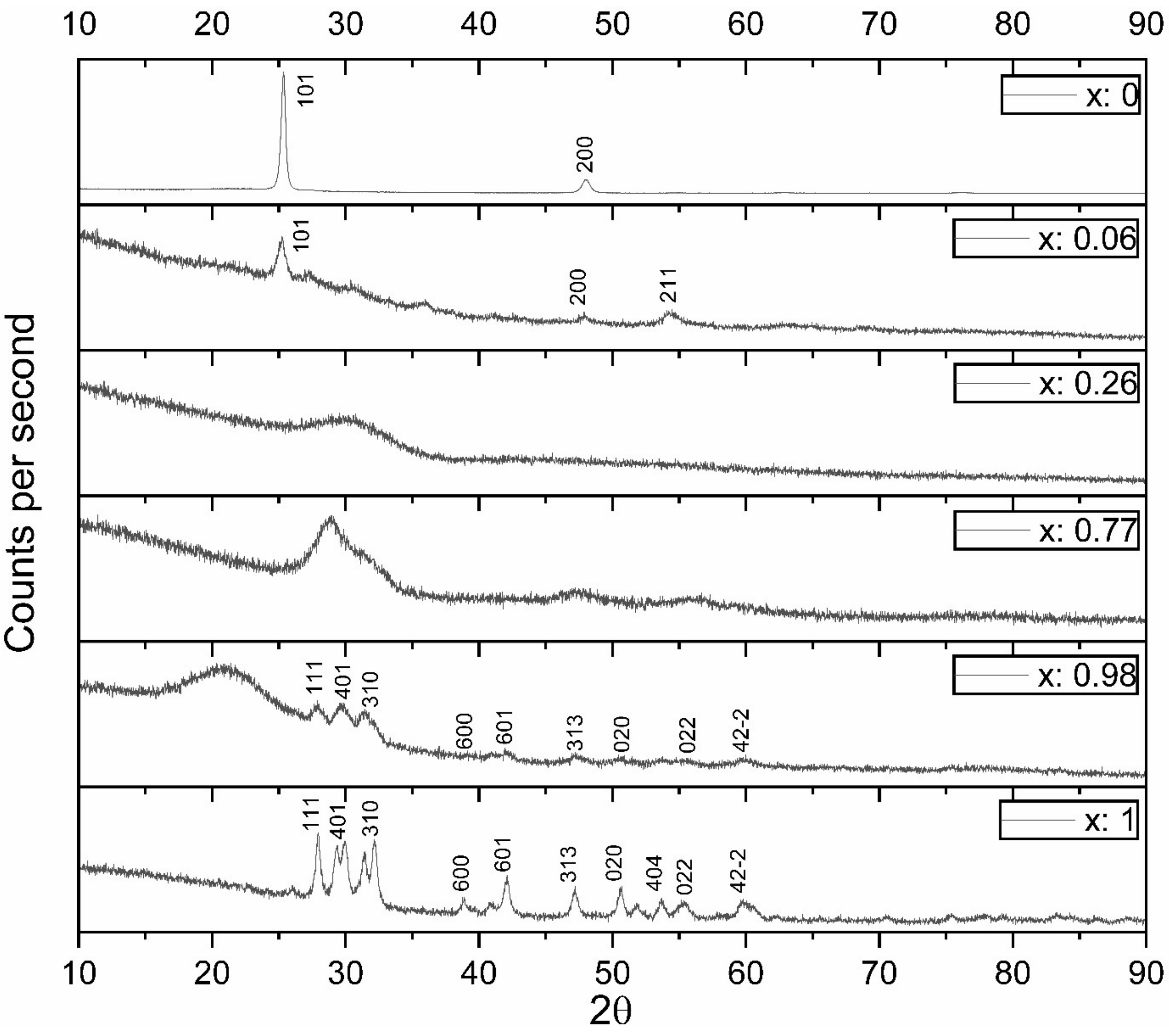

Figure 11 Thin film XRD patterns for the ETO thin films annealed at 600°C with x = 0, 0.06, 0.26, 0.77, 0.98 and 1 Eu/(Eu+Ti).

2.4 Raman spectroscopy

Vibration modes of molecules in the x = Eu/(Eu+Ti) thin films were investigated by Raman spectroscopy (Figure 12 & Figure 13). Various peaks illustrated in the Raman spectra of pure TiO_2 simulated with x = 0.06 after annealed at 600°C (Figure 13) are 144(E_g), 401(B_{1g}), 521(A_{1g}+B_{1g}) and 637 cm^{-1} (E_g) correspond to the Raman active mode. The specific large intensity peak at Raman shift 144 cm^{-1} indicates the anatase phase of TiO_2.[2,3] The pure TiO_2 sample is the least complex compound analysed, which exhibits a strong resemblance between XRD and Raman analyses. The as-deposited thin film consisting entirely of Eu_2O_3 was confirmed to be amorphous, as shown in Figure 12, which displays broad peaks in the Raman spectra and XRD

patterns. After annealing, the thin films with x = 0.98 and 1 exhibit several distinct peaks (Figure 13). These peaks are characteristic of the monoclinic structure of Eu_2O_3, indicating a transition from the amorphous to a more crystalline phase following the annealing process. Various peaks illustrated in the Raman spectra are 111(B_g), 175(A_g), 243(A_g), 282(B_g), 424(B_g) and 583 cm^{-1} (E_g) correspond to the Raman active mode.[4]

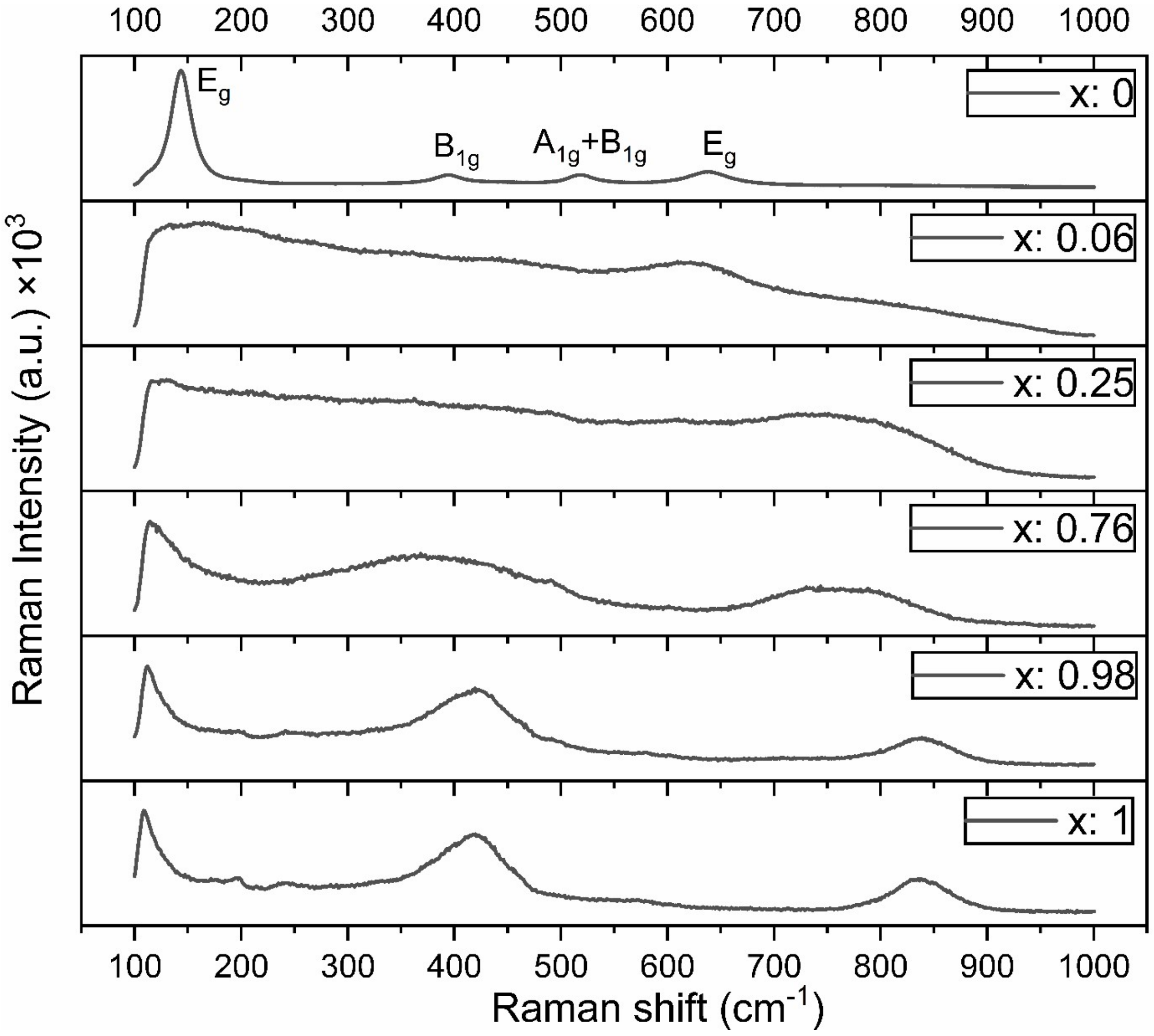

Figure 12 Raman spectra of ETO at x = 0, 0.06, 0.26, 0.77, 0.98 and 1 thin films as-deposited.

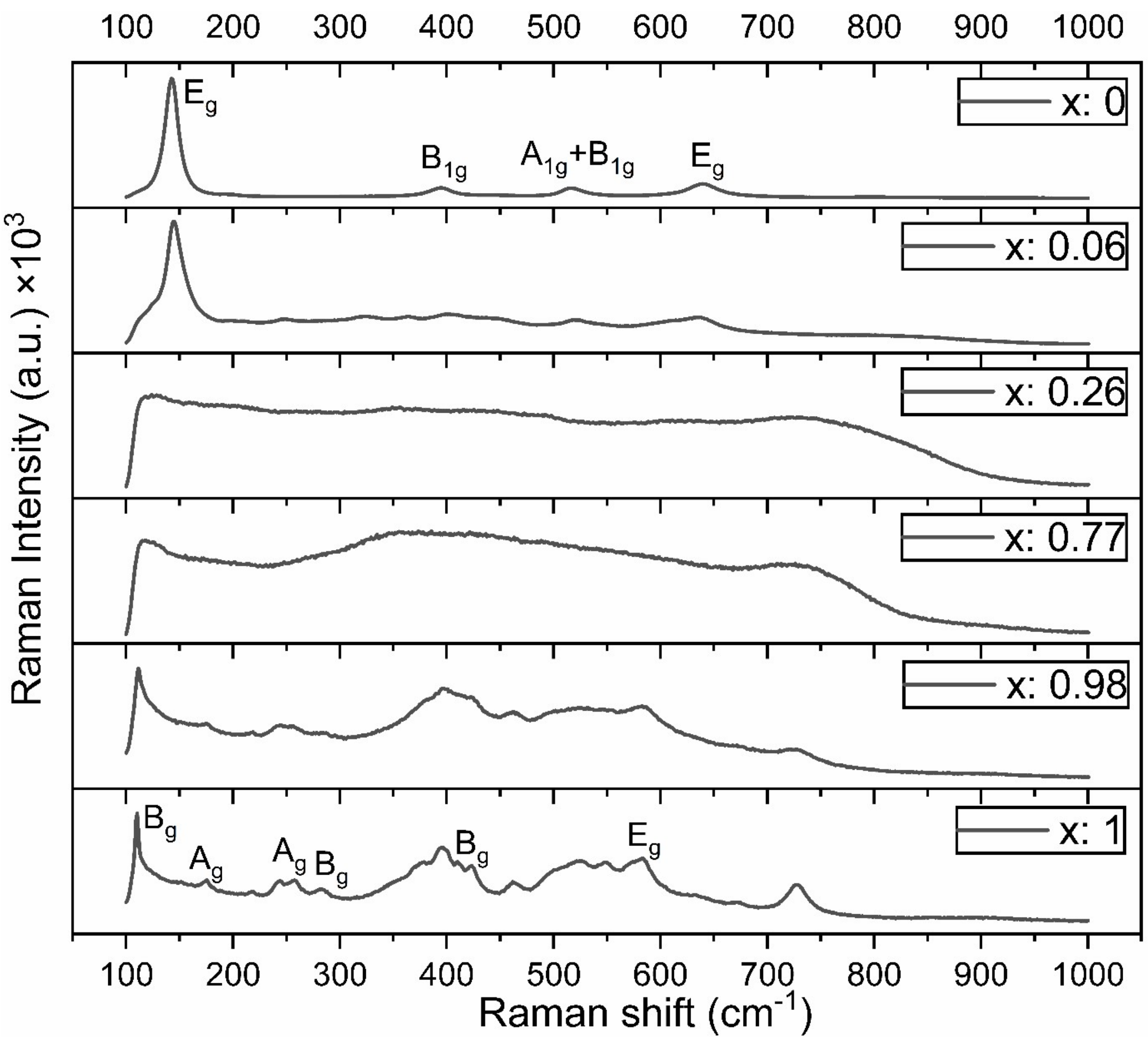

Figure 13 Raman spectra of ETO at x = 0, 0.06, 0.26, 0.77, 0.98 and 1 thin films after annealing at 600˚C.

2.5 Fluorescence of different rates of Europium

2.5.1 Fluorescence of samples As-deposited and after 450˚C and 600˚C annealed

The excitation spectra are measured from 320 to 500 nm with an increment of 1 nm, a slit of 1 nm, and 0.1 s dwell time while fixing the emission wavelength at 613 nm with a 1 nm slit width.

Eu^{3+} ions are excited to the $^5L_6$ level when under the excitation wavelength 394 nm, and a non-radiative energy transfer from the $^5L_6$ level to the $^5D_0$ level for radiative emission. For the excitation spectra (Figure 14 (a) and (b)) with a fixed typical emission

wavelength at 613 nm. Several specific excitation peaks are exhibited in the excitation spectra (320—500 nm) are located at 362, 376, 382, 394, 414 and 464 nm which are attributed to the specific 4*f*-4*f* transition of Eu^{3+} ions corresponding to $^7F_0 \rightarrow {}^5D_4$, $^7F_0 \rightarrow {}^5G_4$, $^7F_0 \rightarrow {}^5G_2$, $^7F_0 \rightarrow {}^5L_6$, $^7F_1 \rightarrow {}^5D_3$ and $^7F_0 \rightarrow {}^5D_2$. Comparing all those transitions in the excitation spectrum, both $^7F_0 \rightarrow {}^5L_6$ and $^7F_0 \rightarrow {}^5D_2$ have strong intensity shown in the spectra of x = 0.06 and 0.08. There is about a five times improvement of the intensity when the thin films after annealing at 600˚C compared with as-deposited at 394 nm.

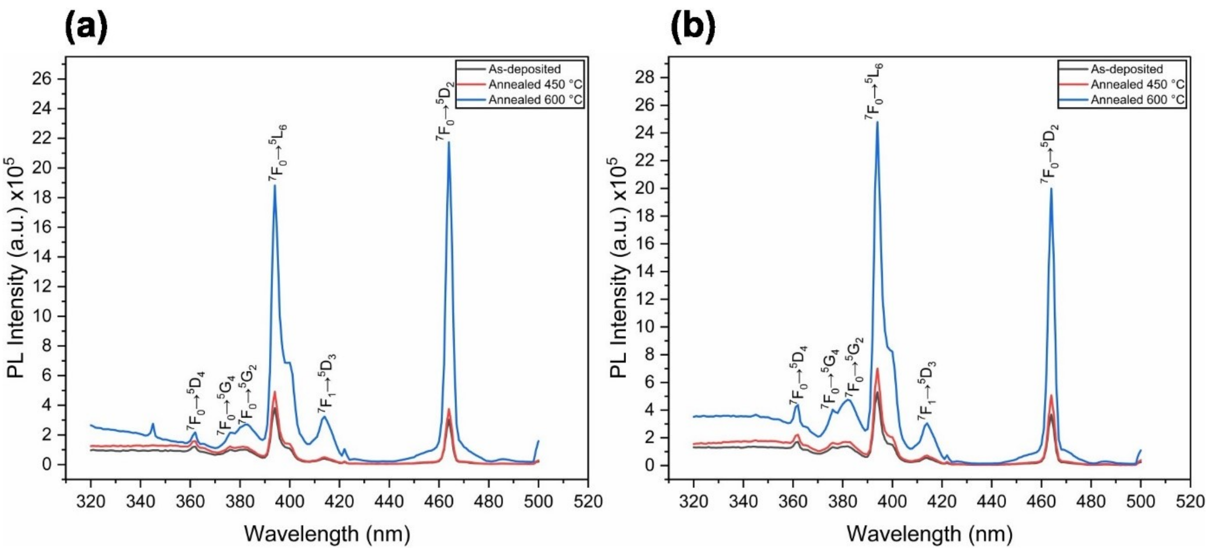

Figure 14 Excitation spectra of thin films with x at (a) 0.06 and (b) 0.08 as-deposition, after annealing at 450˚C and annealing at 600˚C.

The thin films with x = 0.06 and 0.08 as-deposited, after annealing at 450˚C and 600˚C are shown in emission spectra from 550 to 710 nm with a fixed typical excitation wavelength at 394 nm (Figure 15). Several specific emission peaks are exhibited in the emission spectra are located at 579, 592, 613, 625 and 700 nm which are attributed to the specific 4*f* level of Eu^{3+} ions corresponding to $^5D_0 \rightarrow {}^7F_0$, $^5D_0 \rightarrow {}^7F_1$, $^5D_0 \rightarrow {}^7F_2$, $^5D_0 \rightarrow {}^7F_3$ and $^5D_0 \rightarrow {}^7F_4$. The emission intensity at 613nm is raised slightly after annealing at 450˚C and has 5 times improvement when the thin films after annealing at 600˚C compared with the thin films as-deposited.

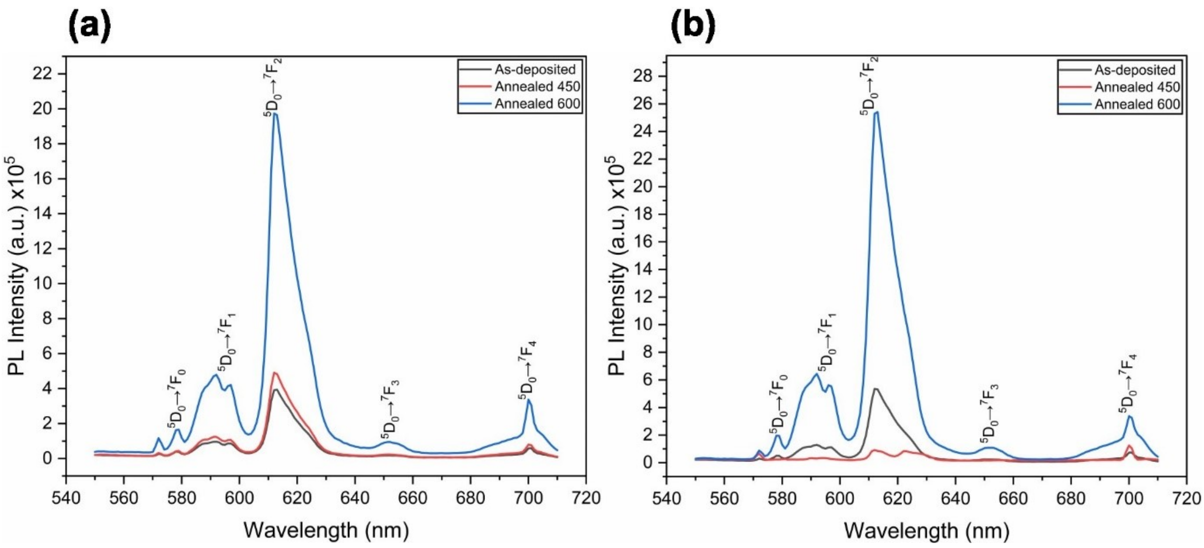

Figure 15 Emission spectra of thin films with x at (a) 0.06 and (b) 0.08 as-deposition, after annealed at 450˚C and annealed at 600˚C.

Several representative thin films with different Eu concentrations are selected to have the fluorescence testing for the comparison, the excitation spectra of thin films with x at 0.05, 0.06, 0.08, 0.18, 0.26, 0.77 and 0.98 with a fixed typical emission wavelength at 613 nm (Figure 16). The transitions $^7F_0 \rightarrow {}^5D_4$, $^7F_0 \rightarrow {}^5G_2$, $^7F_0 \rightarrow {}^5L_6$ and $^7F_0 \rightarrow {}^5D_2$ as excitation wavelengths. All these thin films have the same transitions in excitation spectra with different Eu concentrations. The thin film at 0.08 has the maximum intensity of excitation spectra.

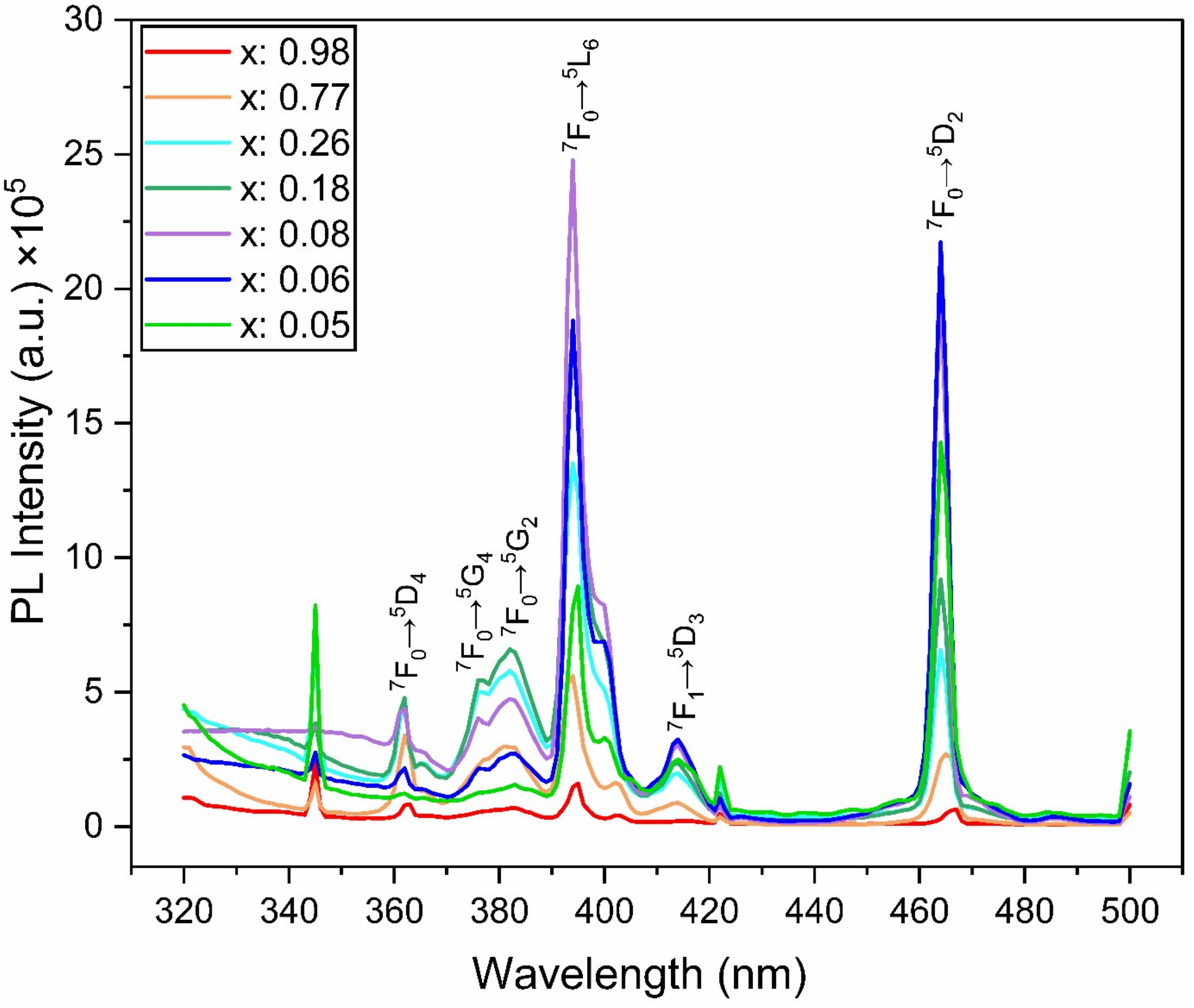

Figure 16 Excitation of x = 0.05, 0.06, 0.08, 0.19, 0.26, 0.77 and 0.98 Eu/(Eu+Ti) samples after annealing at 600˚C, the emission spectrum based on 394 nm excitation wavelengths.

2.6 Transmittance and Bandgap of thin films as-deposited and after 450˚C and 600˚C annealed

UV/Vis was carried out by JASCO V-670 UV-Vis-NIR for the transmittance measurement from 200 nm to 1500 nm. The spot size is 10 × 10 mm, and the data interval is 0.5nm with 400nm/min scan speed in the measurement, with the light source being D2/WI and PTFE-based as the calibration material.

The Bandgaps of the thin films are achieved in the Tauc plot calculated from transmittance spectra via Equation (3). The Tauc plots are drawn by Equation (4). Several representative thin films with different Eu concentrations are selected to have the UV-Vis testing for the comparison, which are x = 0.06, 0.26, 0.77 and 0.98.

Transmittance spectra and Tauc plots included the thin films as-deposited, after annealing at 450˚C and 600˚C (Figure 17). The thin films have high transmittance (T%) from 450 to 1500 nm, and the thin films with x at 0.06, 0.26, 0.77 and 0.98 have about 75T%, 78 T%, 82 T% and 90 T%, respectively. All the samples have sharp absorption edges from 300 to 400 nm, and they are able to absorb the photons in the UV region. Meanwhile, the bandgaps of the thin films are increased with the Eu concentration rise; the bandgaps of x = 0.06, 0.26, 0.77 and 0.98 thin films are 3.33, 3.61, 3.95, and 5.32 eV, respectively, after annealing at 600 ˚C. Transmittance spectra and bandgaps have similar results for the thin films as-deposited, after annealing at 450˚C and 600˚C.

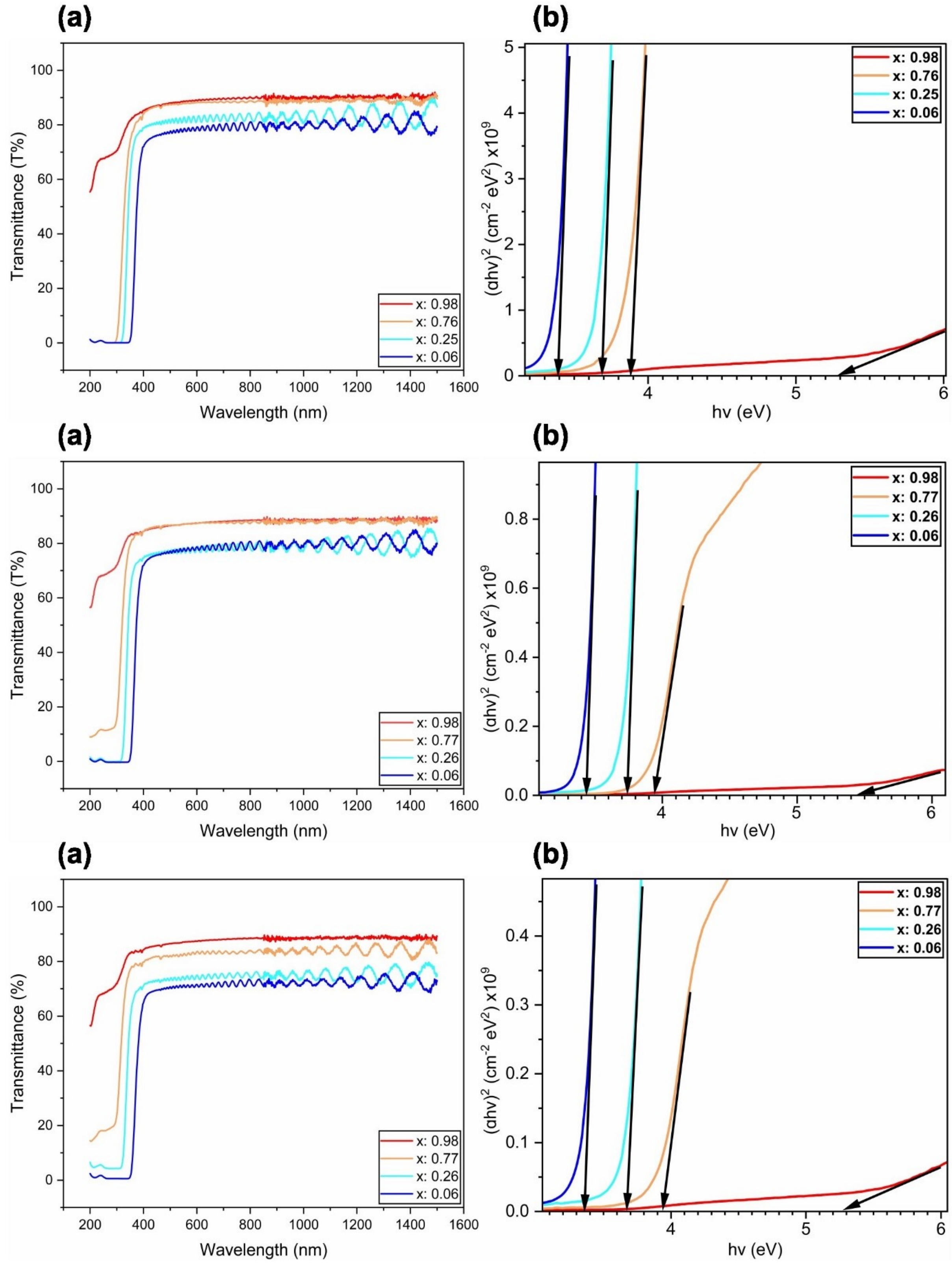

Figure 17 Transmittance and Bandgap of x = 0.06, 0.26, 0.77 and 0.98 thin films (a) & (b) as-deposited, (c) & (d) after annealing at 450˚C, and (e) & (f) after annealing at 600˚C.

The transmittance and bandgaps of the pure Eu_2O_3 and pure TiO_2 thin films after annealing at 600˚C are depicted in the transmittance spectra and tauc plot (Figure 18). These two thin films have higher transmittance from 450 to 1500 nm about 75 T%, but the pure TiO_2 has strong oscillations observed in this region. Pure Eu_2O_3 has sharp absorption edges of around 300 nm, and pure TiO_2 has sharp absorption edges of around 400 nm, which can absorb photons in the UV region. The bandgap of the pure Eu_2O_3 and pure TiO_2 thin films are 3.28 and 3.83 eV, respectively.

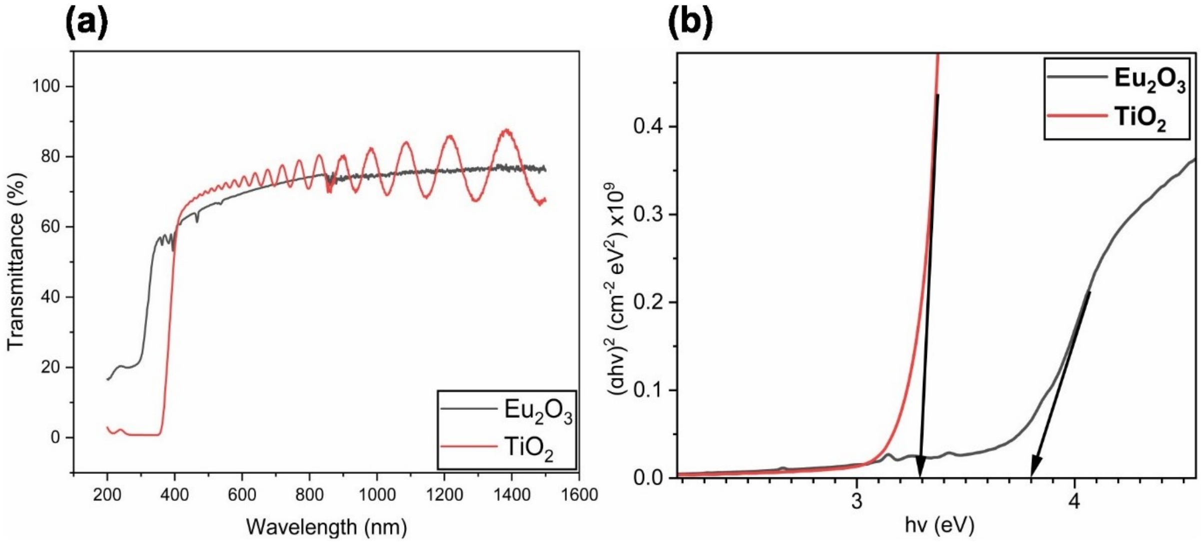

Figure 18 Transmittance and Bandgap of (a) pure Eu_2O_3 and (b) pure TiO_2 thin films after annealing at 600˚C.

The thin film coatings with x below 0.5 were applied to flexible substrates of PET and PDMS, and all these samples were as-deposited without annealing. The PL from the as-deposited samples from x = 0.08 on PET are shown in Figure 19 (a) the flat state and (b) the bent state; the samples from x = 0.44 on PET are shown in Figure 19 (c) the flat state and (d) the bent state. Similarly, the samples from x = 0.08 on PDMS are shown in Figure 19 (e) the flat state and (f) the bent state; the samples from x = 0.44 on PDMS are depicted in (g) the flat state and (h) the bent state. The coatings deposited on flexible substrates have an amorphous phase and exhibit red light PL without annealing when under UV illumination, even when subjected to a degree of bending.

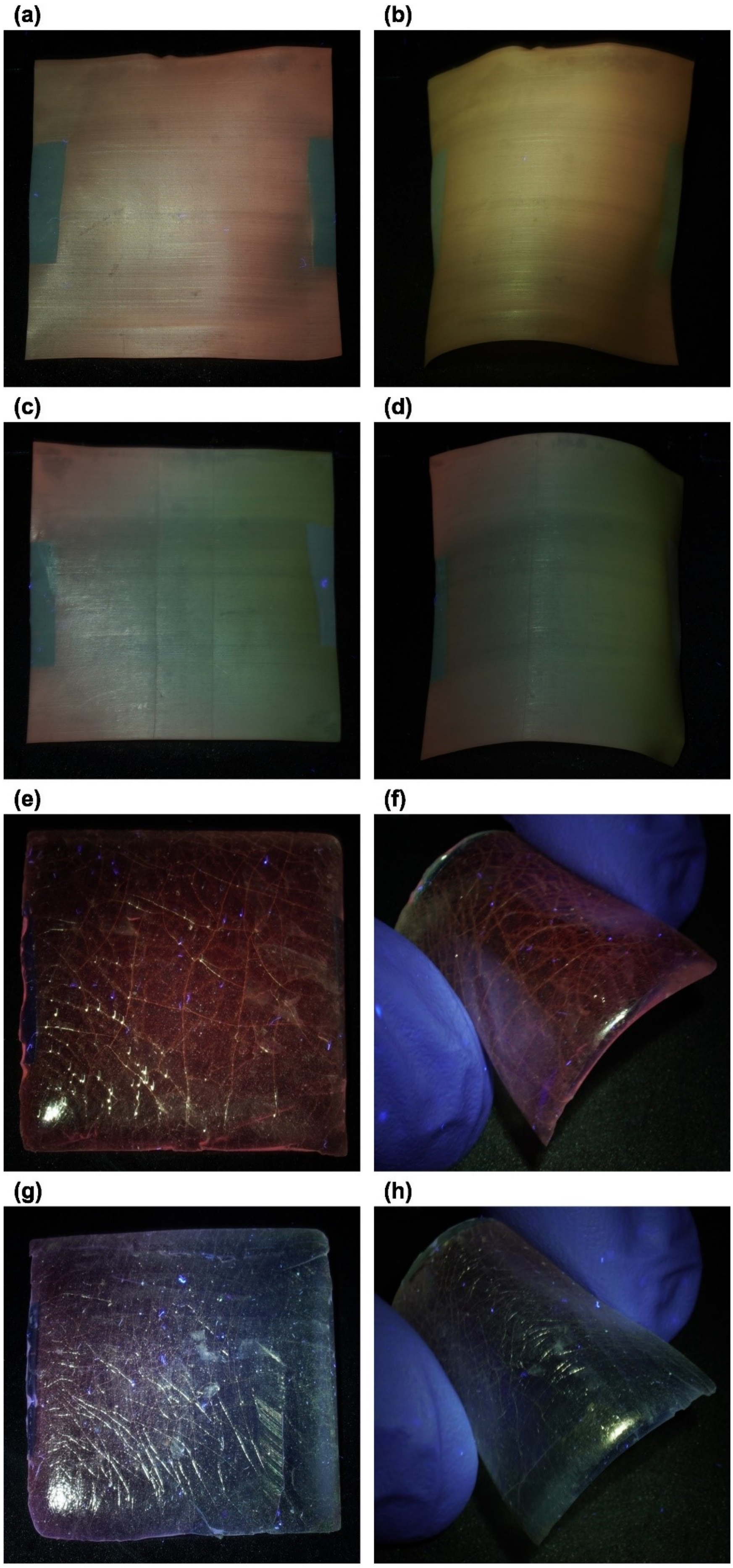

Figure 19 Visual inspection of luminescence thin films as-deposited under 365nm UV light. x = 0.08 on PET (a) flat state, (b) bent state, x = 0.44 on PET (c) flat state, (d) bent state. x = 0.08 Eu/(Eu+Ti) on PDMS (e) flat state, (f) bent state, x = 0.44 on PDMS (g) flat state, (h) bent state.

The PL excitation spectra (Figure 20 (a) and (b)) of ETO thin films were recorded with a fixed emission wavelength at 613 nm, corresponding to the $^5D_0 \rightarrow {}^7F_2$ transition. The excitation peaks can be found at 275 nm, 394 nm, and 465 nm. However, the excitation at 394 nm leads to a much higher emission intensity than that at 280 nm. Consequently, 394 nm was identified as the excitation wavelength for the ETO thin films rather than 280 nm.

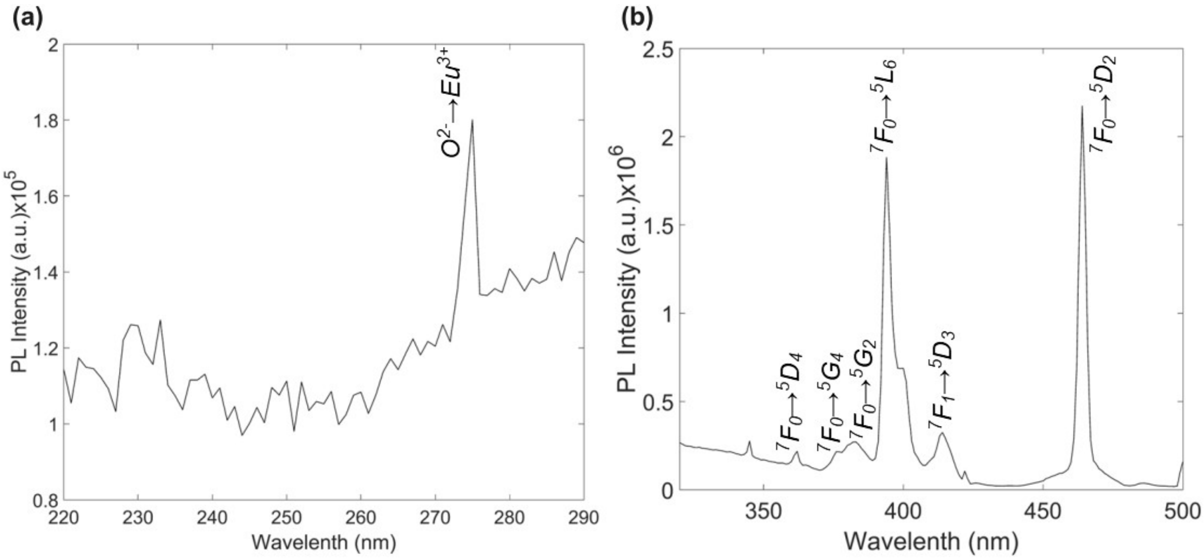

Figure 20 Excitation spectra of thin films with x at 0.06 after annealing at 600˚C in (a) 220—290 nm and (b) 320—500 nm.

3 Reference

1. Sugapriya S., Sriram R., Lakshmi S. Effect of annealing on TiO2 nanoparticles. Optik. November 2013; 124(21): 4971–4975. Available at: DOI:10.1016/j.ijleo.2013.03.040

2. Komaraiah D., Radha E., James J., Kalarikkal N., Sivakumar J., Ramana Reddy M V., et al. Effect of particle size and dopant concentration on the Raman and the photoluminescence spectra of TiO 2 :Eu 3+ nanophosphor thin films. Journal of Luminescence. Elsevier B.V.; 1 July 2019; 211: 320–333. Available at: DOI:10.1016/j.jlumin.2019.03.050

3. Ohsaka T., Yamaoka S. EFFECT OF HYDROSTATIC PRESSURE ON THE RAMAN SPECTRUM OF ANATASE (TiO 2). Solid State Communications.

4. Jiang S., Zhang J., Yan S. Shear stress induced phase transitions of cubic Eu2O3 under non-hydrostatic pressures. AIP Advances. American Institute of Physics Inc.; 1 May 2023; 13(5). Available at: DOI:10.1063/5.0140946
